# Metabolic Bypass Rescues Aberrant S‐nitrosylation‐Induced TCA Cycle Inhibition and Synapse Loss in Alzheimer's Disease Human Neurons

**DOI:** 10.1002/advs.202306469

**Published:** 2024-01-18

**Authors:** Alexander Y. Andreyev, Hongmei Yang, Paschalis‐Thomas Doulias, Nima Dolatabadi, Xu Zhang, Melissa Luevanos, Mayra Blanco, Christine Baal, Ivan Putra, Tomohiro Nakamura, Harry Ischiropoulos, Steven R. Tannenbaum, Stuart A. Lipton

**Affiliations:** ^1^ Department of Molecular Medicine and Neurodegeneration New Medicines Center The Scripps Research Institute La Jolla CA 92037 USA; ^2^ Department of Biological Engineering Massachusetts Institute of Technology Cambridge MA 02139 USA; ^3^ Northeast Asia Institute of Chinese Medicine Changchun University of Chinese Medicine Changchun 130021 China; ^4^ Children's Hospital of Philadelphia Research Institute and Departments of Pediatrics and Pharmacology Raymond and Ruth Perelman School of Medicine at the University of Pennsylvania Philadelphia PA 19104 USA; ^5^ Department of Chemistry and Institute of Biosciences University Research Center of Ioannina University of Ioannina Ioannina 45110 Greece; ^6^ Department of Neurosciences School of Medicine University of California at San Diego La Jolla CA 92093 USA; ^7^ Present address: The Public Experiment Center Changchun University of Chinese Medicine Changchun 130117 China

**Keywords:** Alzheimer's diseases, S‐nitrosylation, tricarboxylic acid cycles

## Abstract

In Alzheimer's disease (AD), dysfunctional mitochondrial metabolism is associated with synaptic loss, the major pathological correlate of cognitive decline. Mechanistic insight for this relationship, however, is still lacking. Here, comparing isogenic wild‐type and AD mutant human induced pluripotent stem cell (hiPSC)‐derived cerebrocortical neurons (hiN), evidence is found for compromised mitochondrial energy in AD using the Seahorse platform to analyze glycolysis and oxidative phosphorylation (OXPHOS). Isotope‐labeled metabolic flux experiments revealed a major block in activity in the tricarboxylic acid (TCA) cycle at the α‐ketoglutarate dehydrogenase (αKGDH)/succinyl coenzyme‐A synthetase step, metabolizing α‐ketoglutarate to succinate. Associated with this block, aberrant protein S‐nitrosylation of αKGDH subunits inhibited their enzyme function. This aberrant S‐nitrosylation is documented not only in AD‐hiN but also in postmortem human AD brains versus controls, as assessed by two separate unbiased mass spectrometry platforms using both SNOTRAP identification of S‐nitrosothiols and chemoselective‐enrichment of S‐nitrosoproteins. Treatment with dimethyl succinate, a cell‐permeable derivative of a TCA substrate downstream to the block, resulted in partial rescue of mitochondrial bioenergetic function as well as reversal of synapse loss in AD‐hiN. These findings have therapeutic implications that rescue of mitochondrial energy metabolism can ameliorate synaptic loss in hiPSC‐based models of AD.

## Graphical abstract

Using human induced pluripotent stem cell (hiPSC)‐derived neurons and postmortem human Alzheimer's disease (AD) brains, the current study links aberrant S‐nitrosylation of tricarboxylic acid (TCA) cycle enzymes to a block in the TCA cycle, compromising energy production. Treatment with a TCA‐cycle substrate to bypass the block partially rescues mitochondrial bioenergetics and synapse loss, suggesting a future therapeutic avenue for AD.

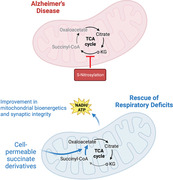

## 1 Introduction

Mitochondrial deficits and bioenergetic compromise have been proposed to contribute to neurodegenerative disorders, including Alzheimer's disease (AD),^[^ ^1^, ^2^, ^3^ ^]^ and have been associated with synaptic loss in human AD brains assessed with specific PET markers.^[^ ^4^ ^]^ Synaptic loss represents a critical neuropathological correlate to cognitive decline.^[^ ^5^, ^6^, ^7^ ^]^ In part accounting for this energy loss, dysfunction of the mitochondrial tricarboxylic acid (TCA) cycle, involving particularly α‐ketoglutarate dehydrogenase (αKGDH, also known as 2‐oxoglutarate dehydrogenase), with some contribution of pyruvate dehydrogenase (PDH), has been identified in several neurodegenerative disorders including AD.^[^ ^1^, ^3^ ^]^ Posttranslational modification of enzymes in the TCA cycle has been identified as an important nidus of control of bioenergetics, but little mechanistic work has been done in this area during disease processes.^[^ ^8^ ^]^ Hence, the underlying mechanism(s) for TCA cycle dysfunction and the full extent of the energy compromise remain unknown. Accordingly, using human postmortem AD brain and hiPSCs to model AD, we report here the presence of aberrant protein S‐nitrosylation of neuronal TCA cycle enzymes, including αKGDH subunits, which inhibits the activity of these enzymes, as we have previously shown.^[^ ^9^, ^10^ ^]^ Recently, we reported the presence of ≈1500 S‐nitrosylation sites on proteins in AD brains and control brains.^[^ ^11^ ^]^ Among the aberrantly S‐nitrosylated proteins in both male and female AD brains compared to controls, we noted that enzymes in metabolic pathways, particularly the TCA cycle, were affected.^[^ ^11^ ^]^ Here, using ^13^C dynamic labeling of AD hiPSC‐derived cerebrocortical neurons (AD‐hiN), we observed specific changes in central carbon metabolism indicating defects in the tricarboxylic acid (TCA) cycle that correlate to the enzymes manifesting aberrant protein S‐nitrosylation. Additionally, using the Seahorse platform, we found that AD‐hiN compared to isogenic WT/Controls manifested a decrease in maximal respiratory capacity (measured as oxygen consumption rate, OCR) but relatively minor effects on basal glycolytic capacity (monitored by extracellular acidification rate, ECAR). These changes were also consistent with TCA cycle dysfunction in the AD‐hiN. In accord with the AD‐hiN findings, the highest level of aberrant S‐nitrosylation in human postmortem brains with AD versus controls was also found in subunits of αKGDH. Thus, our hiPSC‐based model had pathophysiologically relevant changes in the S‐nitrosylation of enzymes in the TCA cycle.

## 2 Results

### 2.1 Protein S‐Nitrosylation of TCA Enzymes in Human AD Brains and AD‐hiN

**Figure 1:**
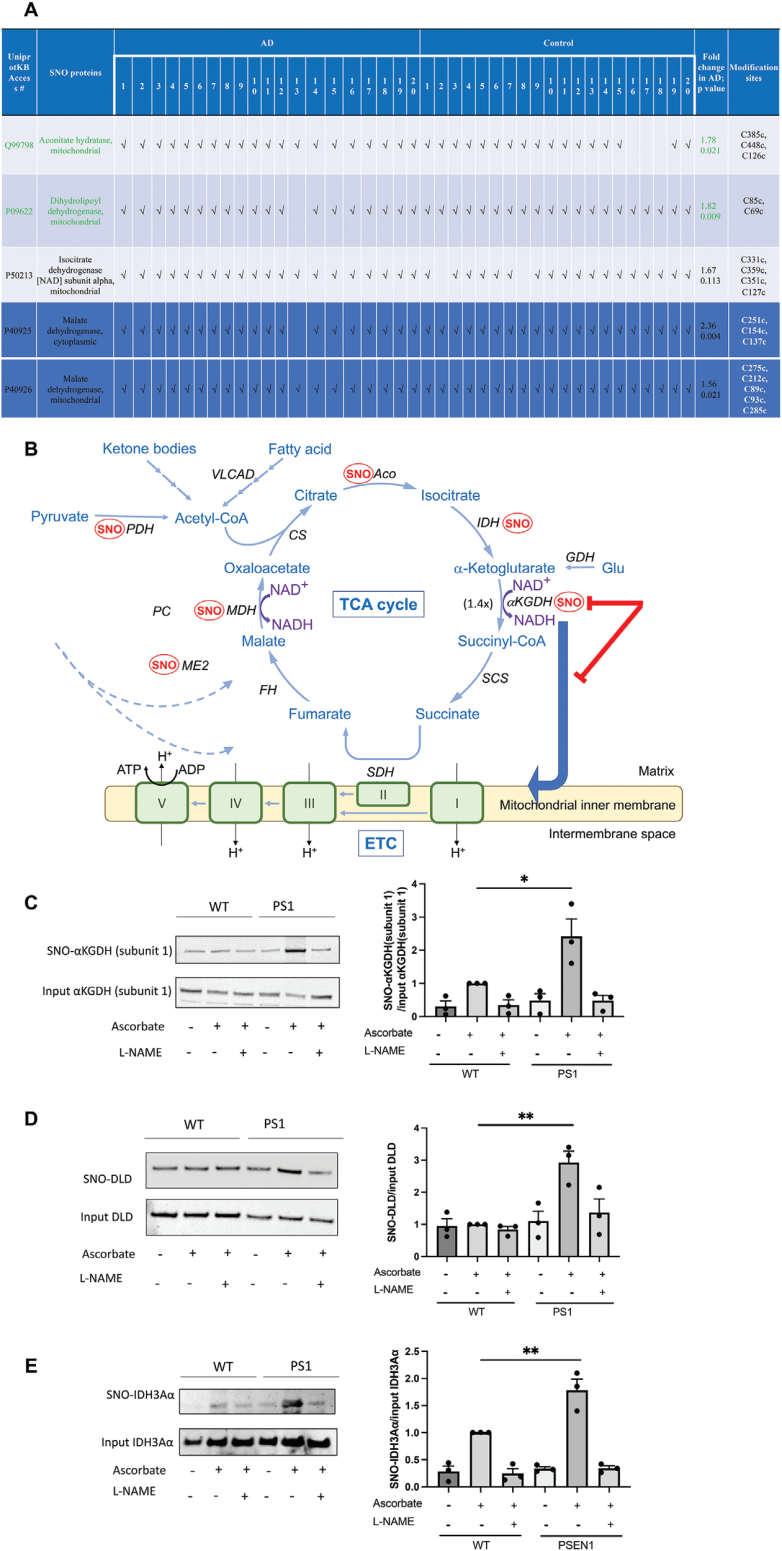
TCA enzymes significantly S‐nitrosylated to a greater extent in AD brains and AD hiN than in controls, as assessed by SNOTRAP/MS and biotin‐switch assays. A) List of S‐nitrosylated TCA enzymes in AD and control brains, assessed as in Yang et al. (2022). The first ten AD and Control brain samples are male, and the second ten in each case are female. Fold increase in AD over Control shown for significant SNO changes among all TCA enzymes with Cys nitrosylation sites, indicated as “modification sites,” as determined by MS. Green values indicate that inhibition in metabolic flux, as determined in isotopic labeling experiments (see Figure 2), was reversed by the NOS inhibitor l‐NAME, consistent with the notion that S‐nitrosylation mediated this inhibition. B) Schema showing effects of S‐nitrosylation (SNO) of TCA cycle enzymes in isogenic WT/Control and AD mutant hiN. AD‐hiN displays basal partial inhibition of the TCA cycle at the Aco/IDH steps. This is consistent with data that at least one of these enzymes is S‐nitrosylated in AD‐hiN (see panel E, below). S‐Nitrosylation of αKGDH results in more major enzyme inhibition, as evidenced by reversal by l‐NAME, and hence significant kinetic inhibition of flux through the TCA cycle at that point in the cycle. The addition of l‐NAME increased the kinetic rate 1.4‐fold at the aKGDH/SCS step in the AD‐hiN, back to that of WT/Control‐hiN, indicating that S‐nitrosylation had slowed the rate by inhibiting enzyme activity. Note that the action of αKGDH also supplies NADH to the ETC for the production of ATP. We have previously performed activity assays showing that S‐nitrosylation of Aco, IDH, αKGDH, and its DLD subunit can inhibit their activity.^[^ ^9^ ^]^ Additionally, inhibition was observed after SNO at the level of PDH, which is S‐nitrosylated via its DLD subunit in AD‐hiN and human AD brain. Aco, aconitase or aconitate hydratase; CS, citrate synthase; DLD, dihydrolipoyl dehydrogenase (subunit 3 of αKGDH and PDH); ETC, electron transport chain; FH, fumarase or fumarate hydratase, IDH, isocitrate dehydrogenase; αKGDH, α‐ketoglutarate dehydrogenase; MDH, malate dehydrogenase (mitochondrial) ME2, malic enzyme 2 (cytoplasmic); PC, pyruvate carboxylase; PDH, pyruvate dehydrogenase; SDH, succinate dehydrogenase (also complex II of the ETC); VLCAD, very long‐chain acyl‐CoA dehydrogenase. C) Biotin‐switch assay to confirm S‐nitrosylation of TCA cycle enzymes in PS1 AD‐hiN versus isogenic WT/Control hiN. hiN cell lysates were subjected to biotin‐switch assay for detection of SNO‐αKGDH subunit 1 and standard immunoblot for total input αKGDH subunit 1. D) hiN cell lysates were subjected to biotin‐switch assay for detection of SNO‐DLD (subunit E3 of αKGDH) and standard immunoblot for total input DLD. (E) hiN cell lysates were subjected to biotin‐switch assay for detection of SNO‐IDH α‐subunit (SNO‐IDH3Aα) and standard immunoblot for total IDH3Aα. For each biotin‐switch assay, a representative blot is shown on the left and a histogram of the densitometry of the bands at the right. The addition of l‐NAME to inhibit NOS or the omission of ascorbate serve as negative controls for the biotin‐switch assay. Data are mean + SEM, *n =* 3, *p < 0.05; **p < 0.01 by ANOVA with Tukey's post‐hoc analysis.

**Figure 2:**
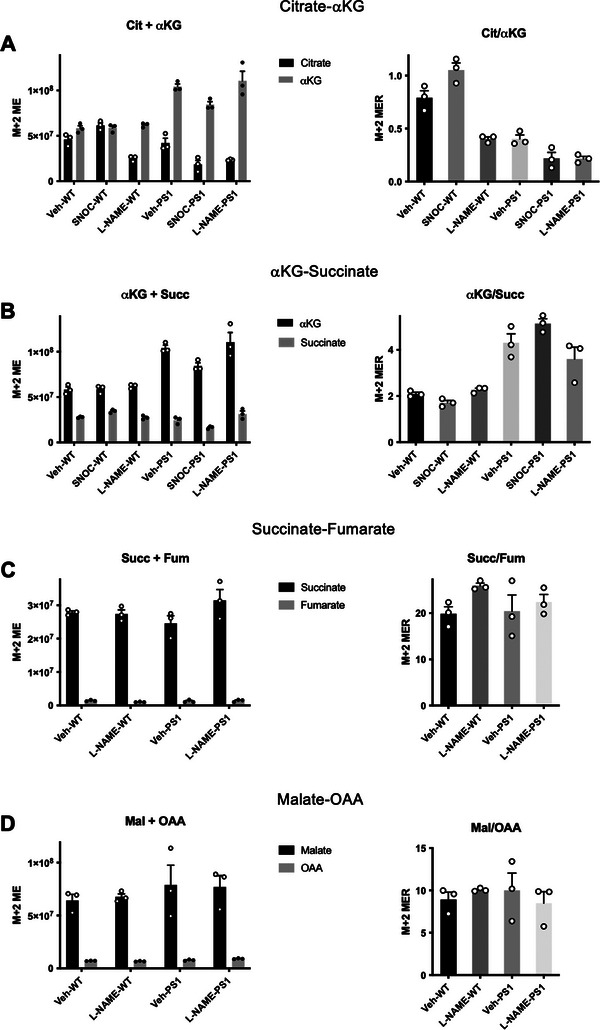
Metabolic flux analysis of TCA cycle enzymes. PS1 AD hiN (versus isogenic WT/Control hiN incubated with ^13^C lactate. Left‐hand panels show molar equivalents of M+2 isotopologues (M+2 ME) of individual metabolites; right‐hand panels show corresponding molar equivalent ratios (M+2 MER) of substrate‐product couples. A) Substrate and product, citrate (Cit) and α‐ketoglutarate (αKG), respectively, of reactions carried out by the aconitase/isocitrate dehydrogenase (Aco/IDH) segment of the TCA cycle. B) α‐KG and succinate (Succ) are the substrate and product, respectively, of reactions carried out by the α‐ketoglutarate dehydrogenase/succinyl coenzyme‐A synthetase (αKGDH/SCS) segment of the TCA cycle. C) Succ and fumarate (Fum) are the substrate and product, respectively, of succinate dehydrogenase (SDH). D) Malate (Mal) and oxaloacetate (OAA) are the substrate and product, respectively, of mitochondrial malate dehydrogenase (MDH). Vehicle (Veh); S‐Nitrosocysteine (SNOC), 100 µM; l‐N^G^‐Nitro arginine methyl ester (l‐NAME), 1 mM. See also Figures S2–S5 (Supporting Information).

Using a novel chemical probe specific for S‐nitrosopeptides called SNOTRAP, we recently used mass spectrometry (MS) techniques for an assessment of the S‐nitrosoproteome in 40 male and female human Alzheimer's brains versus control brains.^[^ ^11^ ^]^ Bioinformatic analysis of these data demonstrated that the TCA cycle enzymes were one of the highest scoring pathways affected by S‐nitrosylation in both male and female AD brains versus controls, and the #2 pathway in male AD brains (as shown in Table S12 and Figure S3, Supporting Information of the prior publication^[^ ^11^ ^]^). By semiquantitative analysis of the SNO‐proteins using spectral counting,^[^ ^11^ ^]^ the most upregulated SNO‐TCA enzymes were aconitase (Aco, also known as aconitate hydratase, *p =* 0.021 by ANOVA) and dihydrolipoyl dehydrogenase (DLD, representing the E3 subunit of α‐ketoglutarate dehydrogenase (αKGDH, also known as 2‐oxoglutarate dehydrogenase) as well as of other dehydrogenases, including pyruvate dehydrogenase (PDH) (*p =* 0.009 by ANOVA) (**Figure** **1**A,B). Malate dehydrogenase, mitochondrial (MDH), was somewhat more S‐nitrosylated in AD brain than in controls (Figure 1A, *p* = 0.021 by ANOVA). Note, however, that malate dehydrogenase, cytoplasmic (also known as malic enzyme 2, ME2) (Figure 1A, *p* = 0.004 by ANOVA), was S‐nitrosylated to an even greater degree in AD than control human brains, consistent with the notion that the alternative anaplerotic pathway access of malate or oxaloacetate into the TCA cycle may be regulated at this level by S‐nitrosylation (Figure 2A).

To confirm the finding of S‐nitrosylated TCA enzymes in human AD brains, we employed a second MS platform in the current study using a chemoselective‐organomercury column to enrich for S‐nitrosylated proteins followed by localization of the SNO‐sites with MS.^[^ ^12^, ^13^ ^]^ The analysis of 3 male AD brains versus 3 control male brains (Table S1, Supporting Information, EXCEL spreadsheet 1, labeled “Human Brains Information”) showed, as expected, that many proteins were S‐nitrosylated differentially in AD brains than in controls (all detected SNO‐peptides and sites shown in Table S1, Supporting Information, EXCEL spreadsheets 2–13 with Venn diagram summary in spreadsheet 9). Interestingly, gene ontology (GO) analysis of biological processes affected by SNO‐proteins showed that TCA cycle enzymes and related metabolic pathways were again among the top hits (Table S1, Supporting Information, EXCEL spreadsheet 13, labeled “GO_BP_AD,” entries highlighted in yellow). In addition to the detection of SNO‐Aco, the chemoselective‐organomercury/MS dataset revealed that the only S‐nitrosylated TCA enzymes that were found exclusively in AD brains and not in Controls were isocitrate dehydrogenase (IDH α‐subunit) and αKGDH subunit 1 (Table S1, Supporting Information, EXCEL spreadsheet 5, labeled “Unique to AD,” highlighted in yellow). Notably, in the initial SNOTRAP/MS dataset, although IDH α‐subunit was also found to be S‐nitrosylated in more AD brains than in non‐AD controls, the mean ratio of spectral counts did not reach significance due to the variance between samples (ratio AD/Control = 1.67, *p =* 0.113 by ANOVA) (Figure 1A). Using the chemoselective‐organomercury/MS approach, SNO‐DLD could not be detected under our conditions so this enzyme could not be compared between the two datasets. Overall, the two detection methods for SNO‐proteins were in agreement for identifying increased S‐nitrosylation of TCA cycle enzymes although some details of the SNO‐sites differed between the two techniques, as might be expected for different methods that complement one another. In the case of the SNOTRAP/MS approach, spectral counts could be used for semiquantitative comparison between AD and Controls to identify SNO‐enzymes in the TCA cycle predominantly found in AD (Aco and DLD, representing subunit E3 of αKGDH) (Figure 1A). For the chemoselective‐organomercury/MS technique, spectral counts for semiquantitative analysis are not available because we do not have multiple peptides for all the proteins, but some TCA SNO‐enzymes (e.g., IDH α‐subunit and αKGDH subunit 1) were found exclusively in AD human brains and not in Control brains (Table S1, Supporting Information, EXCEL spreadsheet 5, labeled “Unique to AD”).

Next, to model the effect of these SNO‐TCA cycle enzymes in a tractable system for mechanistic studies, we compared presenilin 1 (PSEN1 or PS1) mutant AD‐hiN to isogenic WT/Controls. Previously, PS1 mutant or amyloid precursor protein mutant (APP^Swe^) AD‐hiN have been shown to manifest several features of human AD brains, including an increased β‐amyloid (Aβ)42/40 ratio.^[^ ^14^, ^15^, ^16^ ^]^ We showed that these AD‐hiN also manifest a full panoply of glutamatergic receptors by 5 weeks in culture and a hyperactive electrical phenotype compared to isogenic WT/Controls, reminiscent of the hyperactivity seen on EEGs of human AD patients.^[^ ^14^, ^15^ ^]^ Here, also similar to results in AD human brains, by biotin‐switch assay we found the largest increases in AD‐hiN compared to isogenic WT/Controls occurred in SNO‐αKGDH (subunit 1) and/or SNO‐DLD (E3 subunit of αKGDH). (Figure 1C,D, and Figure S1, Supporting Information). Additionally, the IDH α‐subunit was found to be significantly S‐nitrosylated in AD‐hiN over isogenic WT/Controls (Figure 1E). Notably, we have previously demonstrated that the formation of SNO‐TCA enzyme at levels similar to those found here (as judged by the ratio of S‐nitrosylated enzyme to total enzyme) significantly inhibits enzyme activity.^[^ ^9^, ^10^ ^]^ Taken together, these findings suggest that under similar conditions, metabolic flux should be inhibited for these TCA cycle enzymes. Hence, we next performed metabolic flux experiments.

### 2.2 Metabolic Flux Assessment of Defects in TCA Cycle Function in AD‐hiN

Basal neuronal energy is thought to be generated by the TCA cycle after the influx of lactate into neurons that is supplied by astrocytes.^[^ ^17^, ^18^, ^19^ ^]^ However, during periods of intense stimulation, neurons can use glucose directly.^[^ ^20^ ^]^ Nonetheless, the lactate shuttle from astrocytes to neurons is posited to be critical for energy production to support normal synaptic function.^[^ ^21^, ^22^ ^]^ Importantly, irrespective of the source of lactate, recent evidence has shown that for prolonged cognitive loads, the use of lactate may be indispensable compared to glucose, suggesting that prevention of cognitive decline as seen in dementia, is dependent on the TCA cycle.^[^ ^22^ ^]^ To test this in PS1 AD‐hiN versus WT/isogenic‐hiN, we supplied isotopically labeled C^13^‐lactate as the sole source of energy to hiN cultures for metabolic analysis. Our use of isogenic controls minimized the potential obfuscating effect of genetic background.^[^ ^10^, ^14^, ^15^ ^]^

For metabolic flux experiments, hiN were grown in cultures lacking astrocytes to which labeled lactate was added in order to simulate lactate supplied by astrocytes to neurons. Lactate is subsequently taken up by neurons and converted to pyruvate to supply the TCA cycle, whose individual enzyme activity and kinetic rate constants (i.e., flux through the system) can be monitored via monitoring the labeled metabolites,^[^ ^24^, ^25^, ^26^ ^]^ as demonstrated here (**Figure** **2**) and in^[^ ^10^ ^]^ in Figure S1 (Supporting Information). As we and others have previously described, three critical principles are employed in interpreting these metabolic flux data:^[^ ^10^, ^27^ ^]^

1. Inhibition of a specific enzyme results in an increase in ^13^C‐labeled substrate and a decrease in ^13^C‐labeled product, resulting in an increased ratio of substrate/product, as measured in lysates by liquid chromatography‐mass spectrometry (LC‐MS) of TCA metabolites.
2. Relief of enzyme inhibition results in a decrease in the ratio of substrate to product back toward baseline. Here, to test for the effect of the reversal of protein S‐nitrosylation on relieving blocks in enzymatic activity, we used the nitric oxide (NO) synthase inhibitor, l‐N^G^‐nitro arginine methyl ester (l‐NAME). l‐NAME prevents generation of reactive nitrogen species (RNS) related to NO that are known to be dramatically increased in AD brain and hiN in response to oligomeric Aβ^[^ ^28^ ^]^ and mediate aberrant protein S‐nitrosylation of critical cysteine residues in or near active sites of multiple enzymes that affect metabolic flux through the TCA cycle.^[^ ^9^, ^10^, ^29^ ^]^ Additionally, the converse is also true – exposure to an NO donor/transnitrosylating agent such as S‐nitrosocysteine (SNOC) should increase protein S‐nitrosylation of TCA enzymes.^[^ ^9^, ^29^ ^]^
3. For C^13^‐lactate supplying the TCA cycle, the first two ^13^C‐labeled carbons in the TCA enzyme substrates and products (labeled M+2) are likely supplied to the TCA cycle directly by lactate via one turn through the cycle. In contrast, if additional carbons are considered, they may come from ancillary pathway metabolism (as shown in Figure S1, Supporting Information of our prior publication^[^ ^10^ ^]^).^[^ ^24^, ^25^, ^26^, ^30^, ^31^, ^32^, ^33^ ^]^ The data shown here represent raw data, after correction for background atmospheric C^13^ and normalization for mass, labeled as M+2 ME (molar enrichment) for each substrate and product (Figure 2, left‐hand panels). The ratio of substrate/product is also shown, labeled M+2 MER (Figure 2, right‐hand panels). Total ME and MER data for each pair of metabolites is shown in Figures S2–S5 (Supporting Information).

### 2.3 Inhibition of Metabolic Flux At the Level of Aconitase and Isocitrate Dehydrogenase

Looking at the citrate (Cit) to α‐ketoglutarate (αKG) ratios (Figure 2A, right‐hand panel), the metabolic flux experiments show the ratio decreases in WT/Control‐hiN after exposure to l‐NAME, consistent with basal inhibition by S‐nitrosylation at the level of Aco/IDH that is relieved by NO synthase inhibition (Figure 2A, right‐hand panel, *p =* 0.0001). In fact, in accord with these results, in both control human brains and in WT/Control‐hiN, some level of basal S‐nitrosylation was observed for Aco and/or IDH (Figure 1A,E, and Table S1, Supporting Information, EXCEL spreadsheet 6, labeled “Shared Proteins and Sites,” lines 15–17 and 164–168). As expected, exposure to SNOC, which would further increase the level of S‐nitrosylation, further enhanced the Cit/αKG ratio (Figure 2A, right‐hand panel, *p* = 0.0025).

Interestingly, for AD‐hiN compared to WT/Controls, the Cit/αKG ratio was decreased (Figure 2A, right‐hand panel, *p =* 0.0001). Inspection of the individual Cit and /αKG levels revealed the explanation for this (Figure 2A, left‐hand panel), as the difference was mainly in the increase in αKG level (*p <* 0.0001), with virtually no change in the Cit level in AD‐hiN compared to WT/Controls. This finding is best explained by the presence of a more prominent block downstream in the TCA cycle in the AD‐hiN, as discussed below. In this case, l‐NAME would further decrease the Cit/αKG ratio in AD‐hiN, (Figure 2A, right‐hand panel, *p =* 0.0189), as the smaller block at the level of Aco/IDH would be relieved. The fact that SNOC also decreased the Cit/αKG ratio somewhat in AD‐hiN (Figure 2A, right‐hand panel, *p =* 0.0205) may indicate that the high levels of NO present at baseline in AD‐hiN coupled with the addition of the NO donor may create such high levels of S‐nitrosylating species that another level of block upstream from Aco/IDH develops that limits citrate production, e.g., via inhibition of pyruvate dehydrogenase (PDH, Figure 2A), which contains a DLD subunit that is S‐nitrosylated to a significant degree (*p =* 0.026) in human AD brain over control (Figure 1A).

### 2.4 Inhibition of Metabolic Flux at the Level of α‐Ketoglutarate Dehydrogenase/Succinyl Coenzyme‐A Synthetase

At the next steps in the TCA cycle, i.e., at the level of α‐ketoglutarate dehydrogenase/succinyl coenzyme‐A synthetase (αKGDH/SCS), as reflected in the αKG/succinate (Succ) ratios (Figures 2A and 2B), the effect of AD‐hiN compared to WT/Control was most prominent in inhibiting flux. Increases in Succ and αKG, with an overall increase in the αKG/Succ ratio at this stage in AD‐hiN (Figure 2B, right‐hand panel, *p <* 0.0001) and to an even greater degree after SNOC (*p <* 0.0001), may reflect a relative block at the level of αKGDH/SCS induced by NO‐related species resulting in protein S‐nitrosylation and consequent inhibition of enzyme activity. Consistent with this notion, after the addition of l‐NAME, the ratio of an αKG/Succ was not significantly affected in WT/Control hiN but decreased in AD‐hiN such that it was no longer significantly different from WT/Control + l‐NAME (Figure 2B, right‐hand panel). The latter reflects partial relief by l‐NAME of inhibition from S‐nitrosylation at the level of αKGDH since AD‐hiN were shown to manifest increased S‐nitrosylation of αKGDH subunit 1 and its E3 subunit DLD (Figure 1C,D), similar to that found in AD brains (Figure 1A and Table S1, Supporting Information, EXCEL spreadsheet 5, labeled “Unique to AD”).

### 2.5 Lack of Inhibition of Metabolic Flux at the Level of Succinate Dehydrogenase

Reflecting flux at the level of succinate dehydrogenase (SDH), AD‐hiN and WT/Control hiN displayed similar succinate/fumarate (Succ/Fum) ratios (Figure 2C, right‐hand panel). The minor effect of l‐NAME apparently increasing this ratio in WT/Control hiN did not reach statistical significance. The fact that we found some evidence for S‐nitrosylation of mitochondrial SDH in both control and AD human brains (e.g., Table S1, Supporting Information, EXCEL spreadsheet 6, labeled “Shared Proteins and Sites,” lines 265, 266) suggests that some amount of regulation by S‐nitrosylation might occur at this level, but the finding that l‐NAME had little or no influence on the Succ/Fum ratio argues that the effect is a relatively minor one.

### 2.6 Lack of Inhibition of Metabolic Flux At the Level of Malate Dehydrogenase

Similarly, distally in the TCA cycle, at the level of MDH, we observed no significant changes in the ratio of malate to oxaloacetate (Mal/OAA) when comparing AD‐hiN to WT/Control hiN or when adding l‐NAME to prevent S‐nitrosylation. Interestingly, we did find evidence for S‐nitrosylation at cysteine 93 of mitochondrial MDH in control and AD human brains (Figure 1A and Table S1, Supporting Information, EXCEL spreadsheet 6, labeled “Shared Proteins and Sites,” line 184); note, however, in AD human brains, S‐nitrosylation at cysteine 93 decreased, and a new site at cysteine 212 was detected (Table S1, Supporting Information, EXCEL spreadsheet 8, labeled “Shared Proteins New Sites,” line 20; spreadsheet 10, labeled “Shared Sites *p <* 0.05,” line 10; spreadsheet 11, labeled “TCA Enzymes,” line 17). Nonetheless, the fact that l‐NAME displayed little effect on the Mal/OAA ratio argues that the effect of S‐nitrosylation at these sites on MDH does not significantly affect enzymatic activity in our cell‐based model. This result emphasizes why S‐nitrosylation sites must be evaluated in the context of functional effects on activity for each enzyme and for an effect on flux through the TCA cycle at that step.

In summary, in AD‐hiN inhibition of metabolic flux at the level of αKDGH/SCS was most prominent compared to WT/Control hiN and at least partially reversed by l‐NAME. These findings are consistent with the notion that this is the most prominent site of inhibition in the TCA cycle by S‐nitrosylation in AD‐hiN. A minor inhibitory effect was observed at the level of Aco/IDH in both AD‐hiN WT/Control hiN, indicating that it probably represents a normal regulatory mechanism at this stage of the TCA cycle. Kinetic modeling of the rate constants of the various TCA cycle enzymes (see METHOD DETAILS) confirmed that inhibition of the TCA cycle at αKDGH/SCS was the most prominent step affected by aberrant S‐nitrosylation in AD‐hiN compared to WT/Control‐hiN. Notably, the rate constant for αKDGH/SCS increased significantly for AD‐hiN values after addition of l‐NAME (back to 100% from ≈70% in the AD‐hiN and ≈60% in SNOC‐exposed AD‐hiN relative to the WT/Control‐hiN value), signifying relief of inhibition and consistent with the notion that this was the major site of blockade in the TCA cycle by S‐nitrosylation (Figure 2A).

### 2.7 Mitochondrial Bioenergetics in AD‐hiN versus WT/Control‐hiN Assessed by Seahorse Metabolic Flux Analysis

For an enzymatic deficiency, such as that induced by aberrant protein S‐nitrosylation, to manifest at a physiological level, it must be severe enough to restrict metabolic flux through an entire critically important pathway. In other words, it has to either affect the rate‐limiting step or cause a shift of the rate‐limiting step to the enzyme in question. In the case of TCA cycle enzymes, the primary pathological scenario would be a decrease in the energy‐transducing capacity of mitochondria that negatively affects their ability to produce ATP.

**Figure 3:**
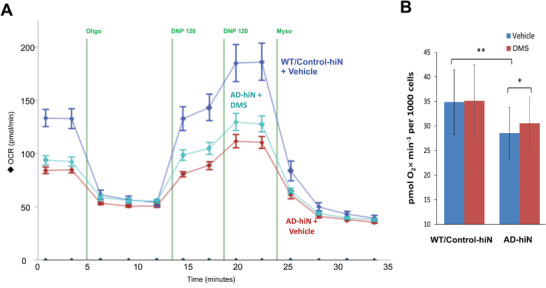
Respiratory defect in PS1 AD‐hiN compared to WT/Control‐hiN and partial rescue with dimethyl succinate. A) Representative experimental run in Seahorse Flux Analyzer with 8‐week terminally differentiated PS1 AD‐hiN and WT/Control‐hiN (10 wells per experimental group). OCR, oxygen consumption rate. Injections shown by vertical lines: Oligo, 2 µg mL^−1^ oligomycin; DNP, 120 µM 2,4‐dinitrophenol; Myxo, 2 µM myxothiazol; DMS, 5 mM dimethyl succinate added 20 min prior to the run. B) Respiratory capacity representing maximal uncoupler‐induced OCR per 1000 cells attained after 4 sequential additions of the uncoupler DNP. Data are mean ± SEM determined in replicate cultures of pure neurons (*n =* 10 wells per group in a single plate of hiN per experiment, with data from 14 separate experiments obtained in separate hiPSC differentiation). *p < 0.05, **p < 0.01 by two‐tailed paired Student's t‐test.

To assess the bioenergetic competence of mitochondria in hiN, we employed the so‐called mitochondrial stress test assay using the Seahorse XF^e^96 metabolic flux analyzer. Interpretation of mitochondrial stress test data and optimized test format are discussed elsewhere.^[^ ^34^, ^35^ ^]^ Briefly, deficits in mitochondrial respiration are critically associated with neuronal dysfunction in AD. Therefore, we evaluated mitochondrial function in both AD‐hiN and WT/Control‐hiN. Using the Seahorse platform to measure OCR, we found a significant deficit in the maximal rate of mitochondrial respiration in PS1 AD‐hiN compared to WT/Control, as assessed after repeated applications of 2,4‐dinitrophenol (DNP), which uncouples OXPHOS from the electron transport chain (ETC) (**Figure** **3**A,B). AD‐hiN manifested a deficiency in spare respiratory capacity of ≈50% compared to WT/Control‐hiN, even when corrected for cell number in the assay (Figure 3B). Both genotypes, however, still possessed some degree of spare respiratory capacity (excess OCR in a maximally DNP‐stimulated state compared to the basal rate at the start of the experiment in Figure 3A). This suggests that the resting/unchallenged AD‐hiN are not energy‐deficient but cannot match the response of WT‐Control hiN to increased energy demand, in this case, mimicked by the uncoupler DNP. In a physiological setting, the ramped‐up energy demand may arise from increased firing rate or synaptic activity.

In the resting as well as the stressed state in AD‐hiN, part of the bioenergetic burden is shifted from functionally challenged OXPHOS to compensatory glycolysis (a Warburg‐like effect), as observed also in AD neurons that have been directly converted from AD patient fibroblasts.^[^ ^36^ ^]^ As an example of this, our ECAR data from the mitochondrial stress test show increased acidification rates in WT/isogenic‐hiN over AD‐hiN, indicative of increased glycolysis in the WT/isogenic‐hiN over the AD‐hiN, consistent with prior findings in mouse AD model systems;^[^ ^2^ ^]^ in the initial basal state, however, ECAR, was higher in AD‐hiN over WT/isogenic‐hiN, indicating some degree of compensation in the AD‐hiN at baseline (Figure S6A, Supporting Information), similar to the conclusions of Traxler and colleagues.^[^ ^36^ ^]^ Assessment of ATP production rates in the basal state, indicative of ATP demand by the cells, shows virtual independence of total ATP demand with respect to genotype (Figure S6B, Supporting Information), with an almost twofold increase in reliance on aerobic glycolysis (Figure S6C, Supporting Information). It should be noted, however, that the contribution of glycolysis compared to OXPHOS remains very minor (≈6% and ≈11% for WT/Control‐hiN and AD‐hiN, respectively).

Further comparing the capacity of glycolysis versus OXPHOS in both the WT/Control‐hiN and AD‐hiN, we found that ATP production was primarily through OXPHOS (i.e., TCA cycle/ETC pathways) rather than glycolysis (Figure S7, Supporting Information). Accordingly, there was a direct correspondence between the lower OCR values of AD‐hiN compared to WT/Control‐hiN (Figure 3B) and the significantly decreased (*p <* 0.01) ATP‐producing capacity of OXPHOS in AD‐hiN versus WT/Control (Figure S7, Supporting Information). Perhaps unexpectedly, the capacity of glycolysis to produce ATP in AD‐hiN was slightly but significantly elevated (*p <* 0.001) compared to WT/Control‐hiN, suggesting that, beyond the mass action‐driven compensatory activation, there is an active upregulation of the pathway in AD‐hiN.

### 2.8 “Substrate Bypass” in the TCA Cycle Results in Improved Bioenergetics in AD‐hiN

Next, we reasoned that by supplying excess substrate to bypass the step in the TCA cycle that was most inhibited via aberrant S‐nitrosylation in AD‐hiN, namely, the production of succinate by αKDGH/SCS, we might be able to improve the overall bioenergetics in these neurons. We, therefore, tested the ability of a membrane‐permeant form of succinate, dimethyl succinate (DMS),^[^ ^37^, ^38^ ^]^ to rescue the respiratory deficiency in AD‐hiN. Importantly, supplying a pro‐drug like DMS that is metabolized to succinate not only provides the substrate to the next enzymatic step in the TCA cycle, SDH, but also to the ETC since SDH also functions as complex II in the ETC (Figure 2A). As predicted, we found using the Seahorse platform that acute application of DMS to AD‐hiN produced partial recovery of respiratory capacity (Figure 3A). Over multiple experiments, the respiratory capacity of the AD‐hiN (both PS1 and APP^Swe^) was ≈20% lower than WT/Control‐hiN, and DMS treatment resulted in recovery of about a third of the deficit (Figure 3B, and Figure S8A,B, Supporting Information). This result represents proof of the principle that bypassing the block in the TCA cycle can rescue the bioenergetics of AD neurons.

As a further indication that DMS enhanced the bioenergetics of AD‐hiN, we monitored mitochondrial NADH using a specific autofluorescence technique (see METHOD DETAILS) and found a relative increase in NADH in AD‐hiN after DMS treatment (Figure S9, Supporting Information). This finding is consistent with the notion that by bypassing the relative block at the αKGDH/SCS step where NADH is produced, additional NADH is generated more distally in the TCA cycle, e.g., at the malate to oxaloacetate step catalyzed by MDH (Figure 2A).

### 2.9 “Substrate Bypass” Partially Rescues Synapses in AD‐hiN

**Figure 4:**
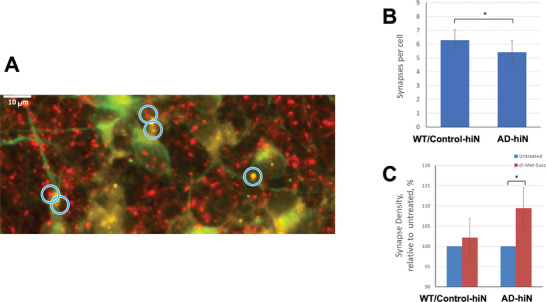
Quantification of synapse number in AD‐hiN and WT/Control hiN. A) Representative field of PS1 AD‐hiN stained for the presynaptic marker Synapsin 1 (red), the postsynaptic marker Homer 1 (yellow), and the neuronal marker microtubule‐associated protein 2 (MAP2, green). Synaptic punctae (blue circles) were identified by co‐localization proximity of pre‐and postsynaptic markers (see METHOD DETAILS). B) Quantification of synaptic punctae per cell in a typical experiment demonstrating ≈14% loss of synapses in the AD‐hiN compared to WT/Control‐hiN. Values are mean ± SEM of 25 separate sets of hiN cultures, each represented by a 10‐well group in a 96‐well plate; *p < 0.05, by Wilcoxon signed‐rank test. C) Dimethyl succinate (DMS, 5 mM) treatment for 48 hr led to the recovery of synaptic density relative to untreated in AD‐hiN but had no significant effect on WT/Control‐hiN. Data are mean ± SEM; *p < 0.05, by Wilcoxon signed‐rank test (*n =* 25 plates assayed).

Loss of synaptic function is considered a primary determinant of AD pathology and cognitive decline.^[^ ^5^, ^6^, ^7^ ^]^ Since the function and maintenance of synapses are energy‐intensive processes important to cognitive activity and the mitochondrial TCA cycle in neurons is critically important in this regard,^[^ ^7^, ^23^ ^]^ even the relatively modest bioenergetic defect observed under stress in AD‐hiN may contribute to synaptic dysfunction and loss. Therefore, we monitored synapse number in cultured AD‐hiN versus WT/Control‐hiN using immunocytochemistry for pre‐and postsynaptic markers with high‐content imaging on the automated ImageXpress Micro Confocal imaging platform (Molecular Dynamics, Inc.). We found a statistically significant deficit in synapse number in AD‐hiN compared to WT/Control hiN (**Figure** **4**A,B), reminiscent of the degree of synaptic loss reported in both transgenic AD mice and human AD brains.^[^ ^5^, ^6^, ^7^, ^39^ ^]^ Notably, treatment of cultures for 48 hr with a cell‐permeable succinate analogue offered statistically significant protection of synapse number in the AD‐hiN (Figure 4C and Figure S8C, Supporting Information). For example, DMS treatment increased synapse density in AD‐hiN ≈70% (calculated from approximately 14% loss of synapses in AD‐hiN compared to WT/Control hiN in Figure 4B, and 9% recovery of synapse number in AD‐hiN with DMS in Figure 4C).

## 3 Discussion

While other publications have suggested a common feature in neurodegenerative disorders of aberrant S‐nitrosylation of TCA cycle enzymes,^[^ ^9^, ^10^, ^29^ ^]^ this is the first study to demonstrate that substrate‐bypass of the major SNO‐induced block in the TCA cycle can at least partially rescue energy supply and, critically, protect from synaptic damage. A recent review emphasized the importance of studying covalent modifications that modulate the TCA cycle.^[^ ^8^ ^]^ In this vein, we describe partial inhibition in flux through the TCA cycle in AD‐hiN caused by aberrant protein S‐nitrosylation prominently at the αKGDH step (subunit 3, DLD, in Figure 1D), which would normally produce NADH and convert αKG to succinate. NADH is thus supplied to complex I of the ETC, and succinate as substrate to SDH, which also functions as complex II of the ETC (Figure 2A). Therefore, inhibition of αKGDH results in both a decrease in substrate for SDH/complex II and also prevents NADH supply to complex I, thus limiting the production of ATP by mitochondria.^[^ ^29^, ^31^, ^32^, ^33^, ^40^ ^]^ In this manner, the formation of SNO‐αKGDH would contribute to the bioenergetic compromise observed in AD‐hiN by Seahorse metabolic flux analysis. Collectively, in both animal models and in hiPSC‐based models, we and others have causally linked such bioenergetic compromise in mitochondria to the pathogenesis of AD and other neurodegenerative disorders by contributing to synaptic loss, and eventually to neuronal cell death.^[^ ^1^, ^35^, ^41^, ^42^, ^43^, ^44^, ^45^ ^]^ The present study links these findings of compromised mitochondrial bioenergetics to TCA cycle compromise, precipitated by aberrant protein S‐nitrosylation reactions observed in human AD brains.

Moreover, our concordant data between chemical S‐nitrosylation in human AD brains and enzymatic inhibition in the TCA cycle in AD‐hiN ‒ for each of the S‐nitrosylation events in TCA cycle enzymes ‒ are consistent with the notion that this posttranslational modification is in large part responsible for the observed inhibition at the αKGDH step of the TCA cycle in human AD brains.^[^ ^1^ ^]^ Furthermore, the fact that the NO synthase inhibitor l‐NAME could reverse the inhibition in metabolic flux monitored with isotope label at the αKGDH step in our AD‐hiN cultures is consistent with the notion that NO‐mediated S‐nitrosylation is responsible for the abnormal inhibitory effect at this step in the TCA cycle that is observed in human AD brains. Of note, other recent findings in another model system using direct conversion of AD patient fibroblasts to neurons have also indicated a Warburg‐like effect with TCA cycle shutdown in AD neurons.^[^ ^36^ ^]^

In terms of potential therapy, when we delivered a membrane‐permeable form of succinate to bypass the relative block at the αKGDH enzymatic step to AD‐hiN, we could partially rescue bioenergetic compromise as assessed by the Seahorse metabolic flux analyzer and, most importantly, restore synapse number. The addition of succinate acts in a dual fashion by increasing the total pool of TCA cycle intermediates (anaplerotic action) and by bypassing compromised complex I of the ETC (as has been reported in AD brains^[^ ^46^ ^]^). Maintenance of even the minimal segment of the TCA cycle metabolizing succinate to oxaloacetate that is not dependent on the activity of DLD‐containing enzymatic complexes (PDH and α‐KGDH), generates one NADH and one FADH_2_, which account for about one‐third of the stoichiometry of the intact TCA cycle. Interestingly, the degree of synaptic loss we observed in our AD‐hiN cultures is similar to the level of loss previously reported in AD transgenic animal models and human AD patients,^[^ ^5^, ^6^, ^7^, ^39^ ^]^ and we could largely rescue this degree of synaptic loss, at least in our AD‐hiN models. This not only serves as proof‐of‐principle that metabolic rescue can affect synaptic number but also suggests future therapeutic avenues that can be pursued for AD, especially in light of the fact that synaptic loss is arguably the best single correlate to cognitive decline in human AD patients.^[^ ^5^, ^6^, ^7^ ^]^

### 3.1 Limitations of the Study

This study is based on findings in postmortem human brains from AD patients and controls. While postmortem times were short (generally a few hours), there is always the possibility that agonal changes could influence the findings. That said, the fact that hiPSC‐based cultures of neurons with AD mutation yielded similar results gives us added confidence in the findings. Moreover, the stress present in these culture systems may cause the neurons to age more quickly,^[^ ^47^ ^]^ and thus provide a reasonable model of neurodegenerative diseases of aging like AD. Nonetheless, using cultured neurons, including AD‐hiN, represents in vitro conditions, and thus informs on what is plausible rather than faithfully replicating in vivo conditions. In the present study, the use of pure hiN in the absence of astrocytes could be viewed as an artificial system. This approach, however, allowed the assessment of human cells from a patient with the disease process and comparison to isogenic, gene‐corrected controls. The fact that several features of AD are faithfully reproduced in these AD‐hiN cultures such as a hyperelectrical phenotype and synaptic damage^[^ ^14^, ^15^ ^]^ gave us increased confidence that aspects of AD such as synaptic loss could be reproduced and studied. Moreover, our finding of similar aberrant S‐nitrosylation reactions in the TCA cycle in fresh postmortem human brain specimens from AD patients and two different (PS1 and APP^Swe^) AD‐hiN supports the pathophysiological significance of our results. Thus, the hiPSC platform was useful for modeling the metabolic and synaptic features of AD. Another critical consideration in the analysis of metabolic experiments is concern over which compartments are being analyzed. In our case, we were able to discern the location of enzymes as mitochondrial versus cytoplasmic via their exact amino‐acid sequences obtained from the MS data for the protein S‐nitrosylation experiments since the mitochondrial and cytoplasmic versions of these enzymes are in general not identical. Moreover, for the metabolic flux experiments, by focusing on the M+2 analysis after heavy isotope labeling of lactate, we limited our conclusions primarily to data obtained after one turn through the TCA cycle in order to focus on events primarily driven by entry into the TCA cycle via lactate and to avoid ancillary pathways of entry. Finally, our use of cell‐permeant succinate derivatives to rescue energy deficits and synapse loss associated with aberrant S‐nitrosylation of upstream TCA cycle enzymes deserves further comment. Off‐target effects of succinate, e.g., to affect the epigenome,^[^ ^48^ ^]^ are possible but unlikely to account for the relatively rapid effects we observed since we also found rapid gains in NADH levels, reflecting improved bioenergetics known to be associated with synapse protection.

## 4 Experimental Section

### hiPSC Cultures

Pluripotent cells (hiPSCs) were cultured and maintained in our laboratory using a protocol described previously.^[^ ^14^, ^15^ ^]^ M146V/WT hiPSC lines bearing the PSEN1 (PS1) M146V mutation or APP Swedish (Swe) mutation and the isogenic WT control (from the laboratory of Marc Tessier‐Lavigne, Rockefeller University/Stanford University, and the NY Stem Cell Institute, NYC). Details regarding these lines were previously published,^[^ ^15^ ^]^ and they were re‐karyotyped to ensure genomic stability. hiPSCs were plated on γ‐irradiated human foreskin fibroblasts and cultured using a medium containing 20% KSR and 8 ng mL^−1^ bFGF, changed daily, as described.^[^ ^14^, ^15^ ^]^ The colonies were manually passaged weekly.

### hiN Neuronal Differentiation

Differentiation of hiPSCs into cerebrocortical neurons (hiN) and detailed characterization of these cultures were performed, as we have described previously.^[^ ^14^, ^15^ ^]^ Just before differentiation, the colonies were dissociated into single‐cell suspension using accutase. To purify hiPSCs and remove fibroblast feeders, a medium containing dissociated fibroblasts and hiPSCs was placed in gelatin‐coated dishes. After adherence, supernatant containing purified hiPSCs was collected and re‐plated at 4 × 10^4^ cells cm^−2^ on Matrigel (BD)‐coated tissue culture dishes for differentiation. The resulting hiN was present in the absence of an astrocyte feeder layer; this was important so that the addition of isotopically labeled C^13^‐lactate in the metabolic flux experiments mimicked the supply of lactate that would normally occur from astrocytes. For most experiments, an 8‐week terminally differentiated hiN was used.

### Human AD and Control Brains

Two sets of human AD and control brains were used in this study. The detailed analysis of the first set, assessed for S‐nitrosoproteins and SNO‐sites with the S‐nitrosothiol‐specific triaryl phosphine probe, SNOTRAP, coupled with MS, was recently published.^[^ ^11^ ^]^ That study included autopsy‐confirmed human brains with AD (*n =* 10 females and *n =* 10 males) and non‐AD (*n =* 10 females and *n =* 10 males), matched for age, sex, education, and ethnicity.^[^ ^11^ ^]^ In a second set of human brains described here, S‐nitrosoproteins and SNO‐sites were assessed by MS after enrichment on a chemoselective‐organomercury column.^[^ ^12^, ^13^ ^]^ This second analysis included autopsy‐confirmed human brains with AD (*n =* 3 males) and controls (*n =* 3 males), matched for age, sex, education, and ethnicity (Table S1, Supporting Information, EXCEL spreadsheet 1, labeled “Human Brains Information”). In all cases, the brain tissues were sampled from the prefrontal cortex (Brodmann area 10). All brain tissues were obtained from the University of California (UCSD) Medical Center and the San Diego VA Medical Center Brain Bank and were flash‐frozen at postmortem examination. The study was approved by the local Ethics Committee of both medical centers (IRB‐18‐7129). Neurological diagnoses were performed independently by two experienced clinicians in alignment with the consensus criteria for AD and were confirmed neuropathologically by the presence of plaques and tangles in the face of neurodegeneration. All the tissues were stored at −80#x000A0;°C until use.

### Preparation of Human Brain Tissues for Mass Spectrometry

Human brain tissues were homogenized on ice using a Teflon pestle and a Jumbo Stirrer (ThermoFisher) in freshly prepared lysis buffer (100 mM HEPES‐NaOH, pH 7.7, 1 mM EDTA, 0.1 mM neocuproine, 1% Triton X‐100, 20 mM IAM, 1% protease inhibitor cocktail, and 0.1% SDS). Homogenates were then centrifuged at 16 000 g for 15 min at 4 °C, and supernatants were collected. Protein concentration was determined by the Bradford assay. One volume of 50 mM HEPES buffer (pH 7.7) was added to the supernatants, which were then centrifuged at 5000 g for 25 min at 4 °C using 10 K MWCO spin filters. The preparation was then stored at −80 °C prior to use.

### Assessment of S‐Nitrosylation Sites in Human AD and Control Brains

For the assessment of the S‐nitrosoproteome in human brains using SNOTRAP labeling followed by MS, we used protocols that we recently published along with their results.^[^ ^11^ ^]^ In the present manuscript, the S‐nitrosylated TCA enzymes in these brains were analyzed. In brief, SNOTRAP‐labeling stock solutions prepared in acetonitrile (ACN) were added to samples at a final concentration of 1.5 mM to selectively convert SNO to stable disulfide‐iminophosphorane. For negative controls, the same volume of 40% ACN was doped in 50 mM HEPES (pH 7.7). The samples were incubated at RT for 2 h. After the reaction, excessive reagents were removed with three washes of 50 mM HEPES, pH 7.7 buffer with 10 K MWCO filters, followed by trypsin digestion overnight at 37 °C.

After enzymatic digestion into peptides, 200 µL Streptavidin agarose beads were added to each sample and incubated for 2 hr at RT with gentle shaking. To minimize non‐specific binding, the beads were washed twice with 5‐fold volumes of the following buffers in succession: Buffer I (100 mM NH_4_HCO_3_ (ABC), 150 mM NaCl, 1 mM EDTA, pH 7.4, containing 0.05% SDS, 0.1% Triton X‐100); buffer II (100 mM ABC, 150 mM NaCl, 1 mM EDTA, pH 7.4 containing 0.1% SDS); buffer III (100 mM ABC + 0.05% SDS + 150 mM NaCl), buffer IV (100 mM ABC), and buffer V (50 mM HEPES, pH 7.7). Peptides bound to beads were then eluted with 10 mM TCEP (in 50 mM HEPES, pH 7.7) and alkylated with 100 mM NEM for 2 h at RT. After alkylation, samples were desalted with Pierce C18 spin columns and stored at –80 °C prior to analysis. Sample preparation and storage were conducted in the dark.

The desalted peptides, dissolved in 0.1% FA, were then analyzed using a Q Exactive MS coupled to Easy‐nLC 1000 (ThermoFisher Scientific, Waltham, MA) interfaced via a nanoSpray Flex ion source. Five technical runs were conducted for each biological replicate, i.e., from the tissue of a single individual. Formic acid in water (0.1%) and in ACN (0.1%) were used as mobile phases A and B, respectively. Aliquots (3 µL) of the samples were injected onto a C18 pre‐column (75 µm ID × 20 mm, 3 µm, Thermo Scientific) and separated by a C18 column (75 µm ID × 250 mm, 2 µm, Thermo Scientific) with a stepwise gradient (1% B for 10  min, 1–60% B for 110  min, and then 60–100% B for 10  min) followed by a 10 min post‐run at 1% B at a flow rate of 300  nL mi^−1^n. Mass spectra were acquired in data‐dependent mode using the following settings: spray voltage, 2.2 kV; capillary temperature, 250 °C; S‐lens RF level, 60%; no sheath and auxiliary gas flow; resolution 70 000; scan range 350–1800 Th. The 10 most abundant ions with multiple charge‐states were selected for fragmentation with an isolation window of 2 Th, and a normalized collision energy of 28% at a resolution of 35 000. The maximum ion injection times for the full MS scan and the MS/MS scan were both 100 ms. The ion target values for the full scan and the MS/MS scans were set to 3 × 10^6^ and 1 × 10^5^, respectively. Xcalibur software was used for data acquisition.

For assessment of the S‐nitrosoproteome in human brains using a second approach, organomercury‐chemoselective enrichment of S‐nitrosothiols, a recently described protocol was followed.^[^ ^9^ ^]^ Experimental details for the preparation and activation of columns and the reaction of homogenate with organomercury resin for S‐nitrosocysteine enrichment were presented previously.^[^ ^12^, ^13^ ^]^ For each group, five samples were analyzed with technical duplicates. For column washes, 50 bed volumes of 50 mM Tris‐HCl (pH 7.4) were used, containing 300 mM NaCl and 0.5% SDS. Following this, 50 bed volumes of the same buffer containing 0.05% SDS were used. Next, columns were washed with 50 bed volumes of 50 mM tris‐HCl containing 300 mM NaCl (pH 7.4), 1% Triton X‐100, and 1 m urea. Columns were then washed with 50‐bed volumes of the same buffer containing 0.1% Triton X‐100 and 0.1 m urea, and finally 200‐bed volumes of water. This was followed by on‐column digestion into peptides; for this, columns were initially washed with 10‐bed volumes of 0.1 m ammonium bicarbonate.

Bound proteins were then digested with Trypsin Gold (1 µg/mL) (Promega) that was added in a one‐bed volume of 0.1 m ammonium bicarbonate in the dark for 16 h at room temperature. Next, the resin was washed with 40 bed volumes of 1 m ammonium bicarbonate (pH 7.4) containing 300 mM NaCl, and then 40 volumes of the same buffer without salt, 40 volumes of 0.1 m ammonium bicarbonate, and 200 volumes of deionized water. Bound peptides were eluted by treatment with a one‐bed volume of performic acid in water.^[^ ^12^, ^48^ ^]^ To increase peptide recovery, the column was further washed with one volume of deionized water. The eluted peptides thus obtained were stored at −80 °C overnight and then lyophilized and resuspended in a 300 µL volume of 0.1% formic acid. The peptides in the resuspended solution were put in low‐retention tubes (Axygen) and the volume was reduced to 30 µL by speed vacuum. Peptides were then desalted using Stage Tips (ThermoFisher) and transferred to a high‐performance liquid chromatography (LC) vial for subsequent LC‐tandem mass spectrometry (MS/MS) analysis of SNO‐peptides using an Orbitrap Elite Hybrid Ion Trap‐Orbitrap Mass Spectrometer (ThermoFisher). As previously described,^[^ ^49^ ^]^ the S‐nitrosoproteome was determined by identifying S‐nitrosylated peptides and proteins for each sample group.

Three different biological samples per group were each analyzed in duplicate. The last step for the organomercury‐based site‐specific identification of S‐nitrosylated cysteine residues consists of using mild performic acid to release the bound peptides selectively and quantitatively. Importantly, the performic acid oxidizes the cysteine thiols to sulfonic acid, thereby generating a unique MS signature that permits site‐specific identification of the modified cysteine residues as >96% of cysteine‐containing peptides were detected with the sulfonic acid modification in the 20 samples analyzed. For the final reporting table, all peptides detected in the duplicate samples for each condition were grouped.^[^ ^50^ ^]^ Note that the organomercury‐chemoselective method identifies the sites of S‐nitrosylation by detecting peptides with modified cysteine residues. Since, like other protein posttranslational modifications, S‐nitrosylation was sub‐stoichiometric, some peptides would not be captured and detected. In this regard, the presence of a particular peptide in one biological condition (e.g., Control brains) and its absence from another could be attributed to the following possible reasons: The cysteine residue was not S‐nitrosylated, or its S‐nitrosylation level was below the detection limit of the method.

### MS Data Processing and Statistical Analysis of Human Brain S‐Nitrosoproteome

For assessment of the S‐nitrosoproteome analyzed by the SNOTRAP/MS method, Agilent Spectrum Mill MS proteomics Workbench B.06 was used for peak list generation, database searching, label‐free semiquantitative assessment, and false discovery rate (FDR) estimation. Parameters for data extractions were as follows: Precursor MH^+^ 300 – 8000 Da, scan time range 0–200 min, sequence tag length > 1, merge scans with same precursor *m/z* ±30s and ±0.05 *m/z*, default for precursor charge, true for find ^12^C precursor, MS noise threshold 100 counts. MS/MS spectra were searched against the human SwissProt protein database (downloaded on 06/18/2019) with ±10 ppm precursor ion tolerance and ± 20 ppm fragment ion tolerance. The search included variable modifications of methionine oxidation, protein N‐terminal acetylation, deamidation of asparagine, and fixed modification of N‐ethylmaleimide on Cys. For both peptide identification and protein polishing, the FDR was set to 1%. Peptide identifications were accepted only if the following confidence thresholds were met: Minimal peptide length was set to five amino acids, and a maximum of two missed cleavages was allowed. The MS/MS spectra were inspected manually to validate the peptide/protein identifications. In addition, for proteins detected in one group but not another, the raw LC/MS data files for the latter were searched manually for the presence of protein‐related peptides detected in the former. The MS raw data were deposited in the ProteomeXchange Consortium (http://proteomecentral.proteomexchange.org) via the PRIDE partner repository with the dataset identifier PXD042436.

For S‐nitrosoproteins identified in the brain with the SNOTRAP/MS technique, the precursor intensities of SNO‐proteins, estimated in Spectrum Mill by combining precursor intensities of the constituent peptides in MS1 spectra, were used to calculate fold‐change quantification of the common SNO‐proteins detected in AD and Control groups, with fold‐change defined as total‐ion intensity AD sample divided by the total‐ion intensity of respective Control. Statistical evaluation of nanoLC‐MS label‐free differential data was performed after the application of data imputation to reduce the number of missing values. Missing values were replaced by this value according to the local Minimum method.^[^ ^51^ ^]^ A fold change of >2 was considered increased expression, while a fold change of <0.5, decreased expression; these cut‐offs corresponded to a p‐value < 0.05, as we have described previously.^[^ ^52^ ^]^ For comparison of quantifications of SNO‐proteins obtained with Spectrum Mill, an ANOVA followed by a post hoc Fisher's PLSD test for multiple comparisons, with a difference of at least *p <* 0.05 considered statistically significant.

For S‐nitrosoproteins enriched by the organomercury‐chemoselective method, after column digestion to generate peptides and elution of peptides, the trioxidation of SNO‐cysteine residues was used to identify the site of modification. Raw MS data were analyzed with MaxQuant open‐source software using cysteine trioxidation and methionine dioxidation as differential modifications. We report the S‐nitrosylation sites on the peptides/proteins based on the MaxQuant output with assessment of SNO‐site presence or absence in specific proteins in AD versus normal Control brain. In the case of the organomercury‐chemoselective/MS method, spectral counting could not be used because multiple peptides for all the proteins were not available. Therefore, the MS1 peak intensity of a peptide was used to compare between Control and AD brain. However, this comparison did not allow us to report on the most important aspect‐ occupancy of the sites. Occupancy was the fraction of SNO modified to unmodified peptide. Previously it was found that the majority of SNO sites were not occupied by other cysteine modifications after organomercury‐chemoselective enrichment;^[^ ^12^, ^13^ ^]^ thus, occupancy only to unmodified peptides was considered.

### Bioinformatic Analysis of S‐Nitrosoproteome Data

To analyze the functional enrichment of gene ontology (GO) processes and pathways for the human AD versus Control brains characterized by SNOTRAP/MS, the SNO‐protein IDs were uploaded into MetaCore software (version 19.4 build 69900). Reactome pathway analysis was performed using STRING (version 11.0) (http://string‐db.org).^[^ ^53^ ^]^ High‐reliability interactions (score > 0.7) were analyzed. To visualize protein‐protein interactions, Cytoscape plug‐in software (version 3.7.2) was used. FDR values <0.1 were accepted for the analyses using the aforementioned software platforms. The gene names for the unique proteins identified for each of the six conditions were submitted to Gene Ontology http://geneontology.org and analyzed for functional processes or cellular component (localization) enrichment.

Following S‐nitrosoproteome identification in human brains by organomercury‐chemoselective enrichment/MS, functional analysis was performed to characterize the biological pathways affected using GO knowledgebase (2020‐12‐08 release) enrichment, pathway analysis using the Kyoto Encyclopedia of Genes and Genomes (KEGG release 96.0, October 1, 2020), and STRING protein‐protein interaction network functional enrichment as described.^[^ ^9^ ^]^

### Biotin‐Switch Assay in hiN for S‐Nitrosylated TCA Enzymes

As previously described, biotin‐switch assays were performed with standard methods.^[^ ^10^, ^41^, ^44^ ^]^ hiN lysates were prepared in HEN buffer [100 mM HEPES‐NaOH (pH 7.4), 1 mM EDTA, 0.1 mM neocuproine] containing 150 mM NaCl, 1% NP‐40, 0.5% deoxycholate, and 0.1% SDS. Next, free thiol groups were blocked with 10 mM S‐methyl methanethiosulfonate (MMTS, Millipore Sigma, 208795) in HEN buffer at 42 °C for 20 min. Following the removal of excess MMTS via acetone precipitation, S‐nitrosothiols were specifically reduced to free thiols using freshly prepared sodium ascorbate (10 mM, Millipore Sigma, 11140). Next, the newly formed free thiols were labeled with 1 mM Biotin‐HPDP(WS) (Dojindo Molecular Technologies, SB17) at RT for 1 hr. The resulting biotinylated proteins were then enriched by binding to High Capacity NeutrAvidin Agarose beads (ThermoFisher Scientific, 29204). Purified biotinylated proteins were then eluted from the beads for immunoblot analysis.

For immunoblots, samples were subjected to Bolt 4–12% Bis‐Tris mini protein gel electrophoresis (ThermoFisher Scientific, NW04122BOX) and transferred to Immobilon‐FL PVDF membranes (Millipore Sigma, IPFL00010). Subsequently, the membranes were blocked using Intercept TBS blocking buffer (Li‐Cor, 927–60001) and then incubated at 4 °C overnight with primary antibodies against αKGDH subunit 1 (Abcam, ab137773), lipoamide dehydrogenase (DLD, Abcam, ab133551), or IDH α‐subunit (ProteinTech, 501730566). Following 3 washes with 1X TBS‐T (Cell Signaling Technology, 9997S), the membranes were incubated with IR‐dye‐conjugated secondary antibody (IR‐dye 800CW‐conjugated goat anti‐rabbit [1:15000; Li‐Cor, 926‐32211]) at RT for 1 hr. Membranes were scanned with an Odyssey CLx infrared imaging system (Li‐Cor). Image Studio software (Li‐Cor) was used for densitometric analysis of immunoblots. Each set of biotin‐switch assays and immunoblots was run on at least three independent biological samples.

### Metabolic Flux and Related Experiments

AD‐hiPSCs and isogenic WT‐hiPSCs were prepared and differentiated into hiN as described above. Two‐thirds of the volume of the culture medium was exchanged biweekly, continuing for ≈5 weeks until experimentation. The last medium change contained 1 mM l‐NAME for some wells for 24 h pretreatment. All wells were then switched for 6 h to Deprivation Medium (± l‐NAME) containing B27/N2 supplements but without glucose/pyruvate/lactate. To ascertain how lactate was handled by the TCA cycle in AD‐hiN versus WT/Control‐hiN, 1 mL of 3X (30 mM) concentrations of either ^12^C or ^13^C‐lactate in Deprivation Medium was added to the appropriate wells. After 6 h labeling, the conditioned medium was harvested and snap‐frozen. Cells were washed once with PBS, solution aspirated, and cells snapped frozen on the plates. Plates and media were then harvested for analysis of labeled metabolites to assess metabolic flux.

Metabolic flux analysis was performed by assessing ^13^C‐labeling of TCA intermediates in the Advanced Technology Core Facility at the Baylor College of Medicine using published protocols,^[^ ^54^, ^55^, ^56^, ^57^ ^]^ building on prior studies.^[^ ^25^, ^30^, ^31^, ^32^, ^33^ ^]^ In brief homogenized samples were extracted in chloroform/methanol/water, and analysis was performed on a 6490 QQQ triple quadrupole mass spectrometer (Agilent Technologies) coupled to a 1290 Series HPLC system via selected reaction monitoring (SRM). Metabolites were targeted in both positive and negative ion modes with an electrospray source ionization (ESI) voltage of +4000 V in the positive ion mode and −3500 V in the negative ion mode. For each detected metabolite, ≈9‐12 data points were collected. For the calculation of labeled metabolites, the equation (Mi*i)/*n =* Mi was used, the ratio of ^13^C‐labeled metabolite to the total pool, where *i* = *M*+*x* (i.e., *M*+2, *M*+3, etc., values) and *n =* number of carbons in the respective metabolite (e.g., for citrate = 6). *M*+*x* values could be summed to yield the molar percent enrichment (MPE), with molar enrichment (ME) equal to MPE times the total pool mean.

### Kinetic Modeling of ^13^C Incorporation into TCA Cycle Intermediates

As described^[^ ^10^ ^]^ using tenets developed by Gupta et al.,^[^ ^57^ ^]^ a semi‐quantitative kinetic model was constructed for analysis of changes in metabolic flux through consecutive enzymatic reactions of the TCA cycle in response to changes in the S‐nitrosylation status of the corresponding enzymes. For multistep segments of the pathway containing unmeasured metabolites, the consecutive reactions were reduced to a single step, containing only measured substrate and product, i.e., the segment between citrate and α‐ketoglutarate was analyzed as if it were a single‐step reaction catalyzed by one‐step enzymatic entity (that combines the activities of Aco and IDH since isocitrate was not measured in the mass spectrometric analysis). Similarly, a two‐step conversion of α‐ketoglutarate to succinate was considered as a single step. The reaction rates (fluxes) were considered to follow mass‐action law kinetics, consistent with the assumption that the substrate concentrations were much smaller in comparison to the corresponding Michaelis constant *K*_m_ for enzymatic reactions for the first turn through the TCA cycle. Accordingly, the flux through each step of the TCA cycle was assumed to be a product of substrate concentration and a corresponding kinetic constant and represent a pseudo‐first‐order reaction process. These assumptions closely follow those utilized in the study by Gupta and colleagues.^[^ ^58^ ^]^

Citrate synthase reaction represents an entry point into the TCA cycle for two‐carbon moieties in the form of acetyl‐CoA (Ac‐CoA), and was, as such, a two‐substrate reaction. A constant level of Ac‐CoA was presumed, as determined by a steady supply of carbons from a virtually unlimited pool of exogenously added lactate (10 mM in the bulk medium). Thus, the flux of the two‐substrate reaction could be reduced to pseudo‐first‐order form with observable kinetic constant *k*_1_ equal to a product of two constants, *k*_1_ = *k*_1_’ × [Ac‐CoA], where *k*_1_’ was the actual kinetic constant for the two‐substrate reaction.

Therefore, our model could be described by six elementary fluxes *J*_i_ = *k*_i_ × [*A*_i_], corresponding to the six measured metabolites (citrate, α‐ketoglutarate, succinate, fumarate, malate, and oxaloacetate). Here, [*A*_i_] was the concentration of analyte number “*i*”, and *k*_i_ was the kinetic constant for the reaction of *A*_i_, with *i* = 1, 2, …6. For each *A*_i_, the rate of change was d[*A*_i_]/d*t* = *J*_i‐1_‐ *J*_i_. For example, malate was the fifth metabolite in the sequence, so the change in malate level d[malate]/*dt* = *J*_4 –_ *J*_5_ = *k*_4_ × [fumarate] – *k*_5_ × [malate]; in other words, the change in malate level equals the difference between malate production from fumarate and malate consumption in malate dehydrogenase reaction.

The initial pools of metabolites were set by a prolonged 6 h starvation period when no source of two‐carbon moieties (i.e., sucrose, pyruvate, nor lactate) was provided to the cells. The assumption was made under these conditions that all TCA cycle metabolites were converted to oxaloacetate and cannot be further converted due to the lack of the second substrate of the citrate synthase reaction (Ac‐CoA). Therefore, the initial condition for simulation was that the level of oxaloacetate equaled the total pool of TCA cycle metabolites, and all other metabolite levels were set to zero; all metabolites were unlabeled prior to the addition of ^13^C‐labeled lactate. Throughout the period of addition, all the entering two‐carbon moieties were considered fully labeled, a reasonable assumption given such a high concentration (10 mM) of labeled lactate was provided.

Simulation of non‐steady state kinetics was performed computationally. The model was applied recursively, starting from the initial conditions, and changing the levels of each metabolite at each recursive step by an increment Δ[*A*_i_]_i_ ≈ *d*[*A*_i_]/*dt* to generate approximate theoretical kinetic curves for isotopologues of each metabolite. This approximation was valid as long as the time increment corresponding to a recursion step was sufficiently small compared to the characteristic time of the total processes. As the interest was only in relative changes upon the biological perturbation triggered by the PS1 mutation in the AD‐hiN in the presence and absence of protein S‐nitrosylation (the absence induced by pre‐treatment with l‐NAME), an arbitrary time scale, satisfying this assumption, was freely chosen. Moreover, the control simulation implied perfectly balanced fluxes throughout all steps. For evaluation of responses to perturbation, the parameters of the model were manually fitted to produce changes in the ratios of consecutive metabolites ([*A*_i‐1_]/[*A*_i_]) within a 3% error of experimentally observed values upon challenge related to nitrosative stress.

### Bioenergetics Assays on hiN with the Seahorse Flux Analyzer

The bioenergetic status of hiN was assessed using a Seahorse XF^e^96 flux analyzer. Human neural progenitor cells were differentiated into hiN in the 60 inner wells of a 96‐well Seahorse cell culture plate for 5–6 weeks at a density of 4 × 10^4^ cells per well (consisting of pure neurons without glial cells). At the start of the experiment, the differentiation medium was replaced with 190 µL assay medium supplemented in treatment well with dimethyl succinate (DMS, 5 mM, Acros). The basal assay medium was custom‐ordered Neurobasal medium (lacking sodium bicarbonate, HEPES, glucose, pyruvate, and Phenol Red); it was supplemented with 10 mM glucose, 20 mM sodium pyruvate, 2 mM GlutaMAX, and 5 mM HEPES‐sodium, pH 7.4. For the mitochondrial stress test assay, replicate readings of basal respiration OCR were followed by injections of ≈10 µL of 10× mitochondrial inhibitors to induce specific metabolic states of mitochondria as follows: The resting state (State 4) was induced by the addition of 2 µg mL^−1^ Oligomycin; maximal respiration (State 3u) was induced by two sequential additions of 90–120 µM uncoupler DNP; and the experiment was terminated by addition of 2 µM of the respiratory inhibitor myxothiazol. Parallel measurements of ECAR were used to estimate the glycolytic contribution to the total energy turnover of the cells. After the assay, cells were fixed with 4% paraformaldehyde for 15 min and stained with Hoechst 33342 (1:500) for subsequent cell counts. For normalization of bioenergetic parameters to cell number, hiN was counted using an ImageXpress Micro Confocal platform, quantifying the central area of each well (inside the “posts”) that contributes to Seahorse‐measured fluxes, and then OCR readings were divided by cell numbers for each well.

### Assessment of ATP Production and Turnover in AD‐hiN and WT/Control hiN

Cellular ATP demand and rates of ATP production were estimated essentially as published previously^[^ ^59^ ^]^ from the OCR and ECAR data using the known stoichiometry between ATP and oxygen and lactate, respectively. ECAR rates expressed in mpH/min reported by the Seahorse XF^e^96 flux analyzer were converted to proton production rate (PPR) expressed as [H^+^]/min. The conversion factor for this was determined by titrating the assay medium, the buffering capacity of which was provided predominantly by the 5 mM HEPES in the medium, with sequential pulses of sulfuric acid (data not shown). The pH response was virtually linear over the working range (6.8 – 7.4) and yielded a conversion factor of 6.07 pmol H^+^ per 1 mpH.

ECAR includes contributions from both lactic acid and CO_2_ in the form of carbonic acid. To determine the activity of the glycolytic (lactate‐producing) pathway, acidification due to CO_2_ produced by respiration had to be subtracted from the total ECAR value. The factor relating change in acidification to respiratory activity was determined empirically by dividing the decrease in ECAR by the drop in OCR upon inhibition of respiration with myxothiazol at the end of the Seahorse run. The product of this factor and OCR was subtracted from ECAR in the basal and/or maximally uncoupled states. The differences, corresponding to glycolytic activities, were converted to PPR using the factor mentioned above; PPR was equivalent to lactate production rate (1:1 lactate to H^+^ given almost complete dissociation at pH 7.4). Since one molecule of ATP was produced per each lactate molecule (1:1 ATP to lactate) in the glycolytic pathway, the rate of ATP production assumes the same value. For ATP production during oxidative phosphorylation, OCR was multiplied by 6, corresponding to the known stoichiometry of 36 ATP per glucose. Both rates were summed up to yield the total ATP production rate.

According to the concept of respiratory control rate of ATP production, in the basal state, this was equal to the demand for ATP by the cells; under normal circumstances, this value would be less than the capacity to produce ATP. The capacity to produce ATP by oxidative phosphorylation or by glycolysis was calculated from the maximal OCR (State 3u) and from the ECAR value in the respiratory‐inhibited cells (determined after the addition of myxothiazol), respectively.

### Analysis of Mitochondrial NADH in AD‐hiN and WT/Control‐hiN

Redox status of pyridine nucleotides in hiN cultures was assessed using autofluorescence (*λ*_ex_ = 340, *λ*_em_ = 460 nm) employing a Zeiss Axiovert100 microscope equipped with a Lambda DG4 (Sutter Instruments) light source, allowing us to monitor relative NADH levels by fluorescence measurement.^[^ ^59^ ^]^ The excitation and emission filters were 340/26 and 470/40 nm (maximal wavelength and bandwidth), respectively. Tetramethylrhodamine, ethyl ester (TMRE, 10 nM) was used as a mitochondrial counterstain (with 560/55 excitation and 655/40 emission). Images were acquired using MetaMorph software (Molecular Devices) and analyzed using Fiji (ImageJ) freeware. Punctate objects representing mitochondria were identified using the Find Maxima function and the intensity of the brightest 200 of them (starting from the 6^th^ brightest to avoid bright artifacts) was measured. In addition to monitoring the NADH at the basal state, reflecting steady‐state conditions of active NADH utilization for ATP synthesis, the resting state induced by 2 µg mL^−1^ oligomycin was analyzed. In the resting state, NADH utilization for ATP synthesis was blocked, and the reduced level of pyridine nucleotides was maximized.

### Cell‐based Quantification of Synapses with Confocal Imaging

For detection of synapses in AD‐hiN and WT/Control hiN, their respective neural progenitor cells were differentiated into cerebrocortical neurons (with no glia) in the 60 inner wells of a 96‐well plate (Corning, #3603) for 5–7 weeks at a density of ≈4 × 10^4^ cells per well (similar to the Seahorse experimental protocol). The cells were treated with 5 mM DMS or vehicle medium for 48 h prior to assessment (10 wells per genotype per treatment were analyzed). hiN were then fixed with 4% paraformaldehyde for 15 min, permeabilized, and blocked with blocking buffer (PBS supplemented with 3% BSA and 0.3% Triton X‐100) for 30 min. The cells were decorated with primary antibodies for Synapsin 1 (rabbit monoclonal, Cell Signaling #5297S, 1:500), Homer 1 (mouse monoclonal, Synaptic Systems #160011, 1:250), and MAP2 (chicken polyclonal, Invitrogen #PA‐10005, 1:500) in the blocking buffer overnight. Cells were washed with PBS and incubated with anti‐rabbit AlexaFluor Plus 647 (Sigma A32795), anti‐mouse AlexaFluor Plus 555 (Sigma A32773), anti‐chicken AlexaFluor Plus 488 (Sigma A32931) secondary antibodies (1:500 in the blocking buffer) for 2 h. The cells were also stained with Hoechst 33342 (1:500) to label nuclei for cell counts.

Images were acquired at 20× magnification in the ImageXpress Micro Confocal platform and analyzed using MetaXpress software (Molecular Dynamics, Inc.) equipped with a custom, automated neurite and synapse detection and quantification module. For synapse detection, pre‐ and postsynaptic terminals were identified in close apposition (≈0.3 µm).^[^ ^61^, ^62^, ^63^, ^64^ ^]^ Pre‐ and postsynaptic terminals were identified as Synapsin 1 and Homer 1‐positive punctae, respectively. Neuronal bodies and neurites were identified by staining for MAP2. Coincidence with MAP2 stain was assessed to ensure the synaptic punctae were associated with neurons. The number of nuclei was counted separately with Hoechst staining and presumed to correspond to cell number. For each well, the number of synapses per cell was calculated. This number was averaged over each 10‐well experimental group for each plate. Alternatively, for some experiments synapse counts were scored manually per unit area using a Nikon Eclipse Ti2 confocal microscope, yielding similar results as the automated imaging with the ImageXpress platform. In addition to dimethyl succinate (DMS), the more cell‐permeable diethyl derivative (DES) was also used in some experiments.

### Quantification and Statistical Analysis

Data were analyzed with the tests indicated in the appropriate paragraph for statistical analysis and bioinformatics (see above). For S‐nitrosylation experiments and metabolic flux data, numbers (*n*) of biological replicates were included in the figure legends; comparisons were made by ANOVA followed by a PLSD or other post‐hoc test.^[^ ^65^ ^]^ For both the analysis of S‐nitrosylated proteins and metabolic flux data, a *p*‐value < 0.05 was considered significant.

## Conflict of Interest

The authors declare no conflict of interest.

## Author Contributions

A.Y.A., H.Y., P.‐T.D., and N.D. contributed equally to this work. Conceptualization, S.A.L.; methodology, A.A., P.‐T.D., H. Y., N.D., S.R.T., H.I., and S.A.L.; investigation, A.A., P.‐T.D., H. Y., N.D., X.Z., M.L., M.B., C.B., and I.V.; visualization, P.‐T.D., H. Y., N.D., A.A., X.Z., and T.N.; funding acquisition, T.N., S.R.T., H.I., and S.A.L.; writing – original draft, S.A.L.; writing – review & editing, P.‐T.D., H.Y., N.D., A.A., X.Z., T.N., S.R.T., H.I., and S.A.L.

## Supporting information

Supporting Information

Supplemental Table 1

## Data Availability

The data that support the findings of this study are available from the corresponding author upon reasonable request.
