# Supplementary material for: Metabolic Bypass Rescues Aberrant S‐nitrosylation‐Induced TCA Cycle Inhibition and Synapse Loss in Alzheimer's Disease Human Neurons
Source: Adv Sci (Weinh). 2024 Jan 18;11(12):2306469. doi: 10.1002/advs.202306469 (PMC10966553; doi:10.1002/advs.202306469)
Supplement: Supplementary file 1 — Supporting Information [file ADVS-11-2306469-s001.pdf]

ADVANCED SCIENCE Open Access

Supporting Information

for *Adv. Sci.*, DOI 10.1002/advs.202306469

Metabolic Bypass Rescues Aberrant S-nitrosylation-Induced TCA Cycle Inhibition and Synapse Loss in Alzheimer's Disease Human Neurons

*Alexander Y. Andreyev, Hongmei Yang, Paschalis-Thomas Doulias, Nima Dolatabadi, Xu Zhang, Melissa Luevanos, Mayra Blanco, Christine Baal, Ivan Putra, Tomohiro Nakamura, Harry Ischiropoulos, Steven R. Tannenbaum and Stuart A. Lipton**

Supplemental Figures, Supplemental Figure Legends, and Supplemental Table Legends

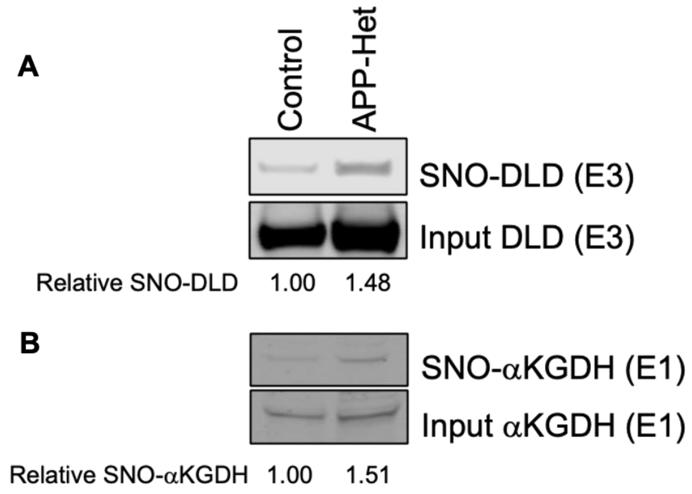

Figure S1. S-Nitrosylation of TCA cycle enzymes in APP^{Swe} AD-hiN vs. isogenic WT/Control hiN

(**A**) Biotin-switch assay to confirm S-nitrosylation of TCA cycle enzymes in APP^{Swe} heterozygous AD-hiN vs. isogenic WT/Control hiN. hiN cell lysates were subjected to biotin-switch assay for detection of SNO-DLD (subunit E3 of αKGDH) and standard immunoblot for total input DLD.

(**B**) hiN cell lysates were subjected to biotin-switch assay for detection of SNO-αKGDH subunit 1 and standard immunoblot for total input αKGDH subunit 1. Relative levels refer to SNO-protein/total protein ratio.

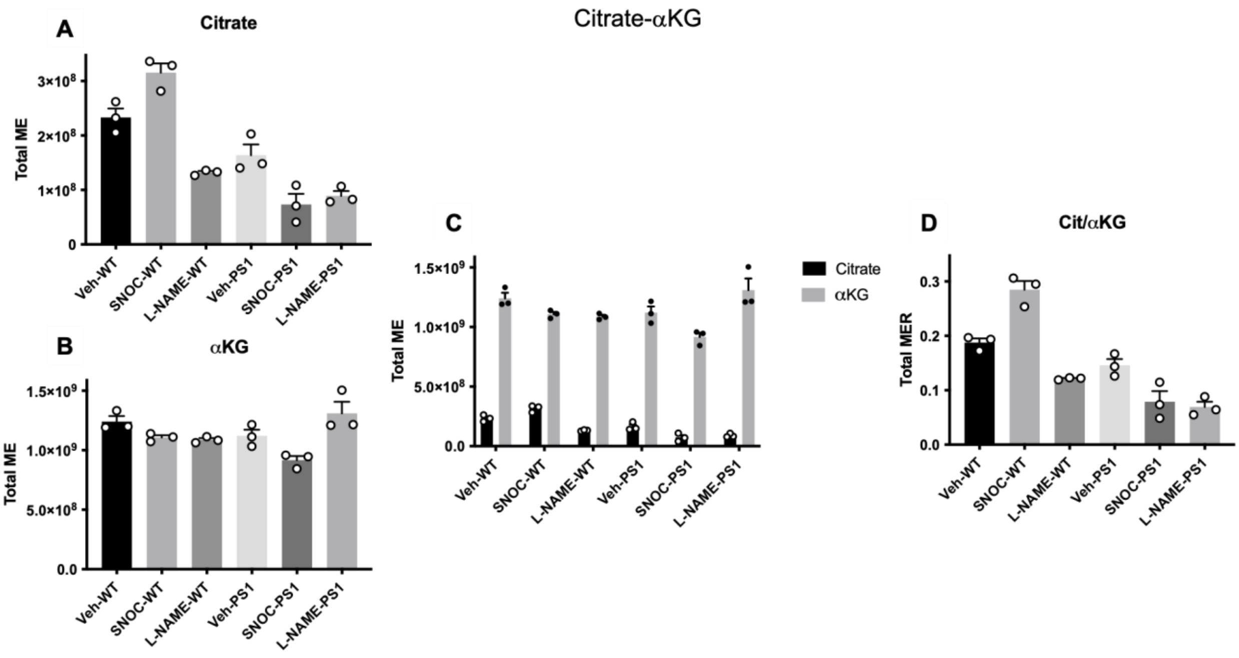

Figure S2. Total ME and MER data for the pair of metabolites citrate and α-ketoglutarate (αKG), representing flux through aconitate and isocitrate dehydrogenase

Values are mean + SEM (n = 3).

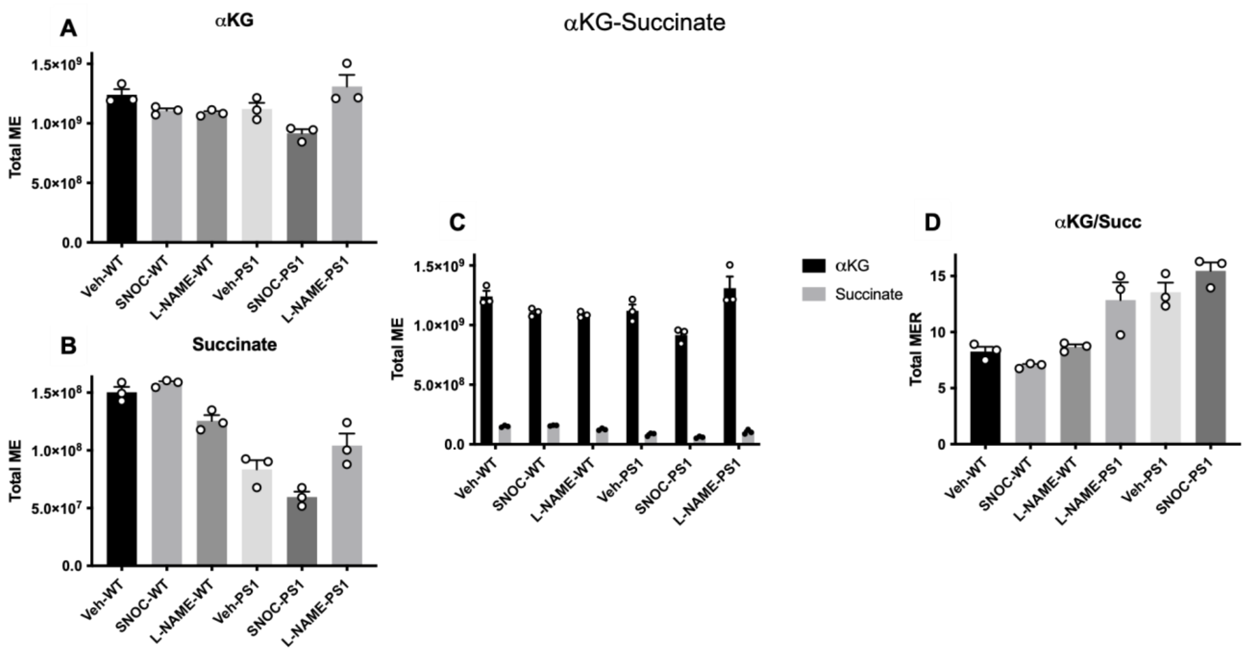

Figure S3. Total ME and MER data for the pair of metabolites α-ketoglutarate (αKG) and succinate (Succ), representing flux through α-ketoglutarate dehydrogenase and succinyl coenzyme-A synthetase

Values are mean + SEM (n = 3).

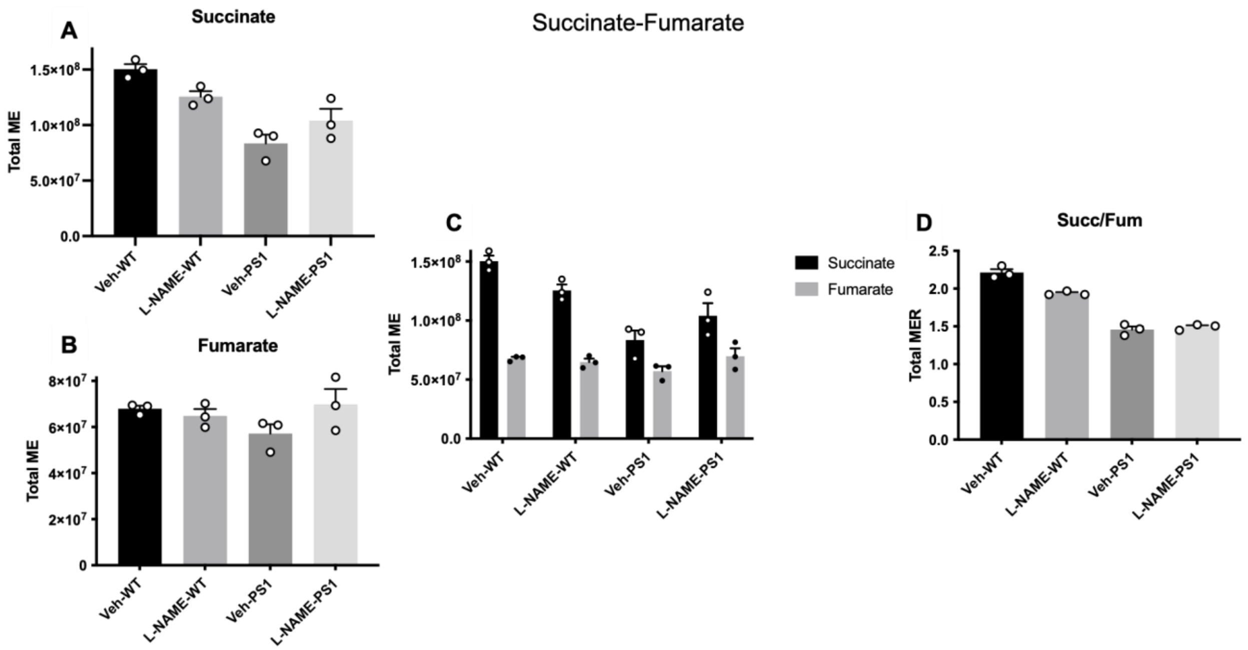

Figure S4. Total ME and MER data for the pair of metabolites succinate (Succ) and fumarate (Fum), representing flux through succinate dehydrogenase

Values are mean + SEM (n = 3).

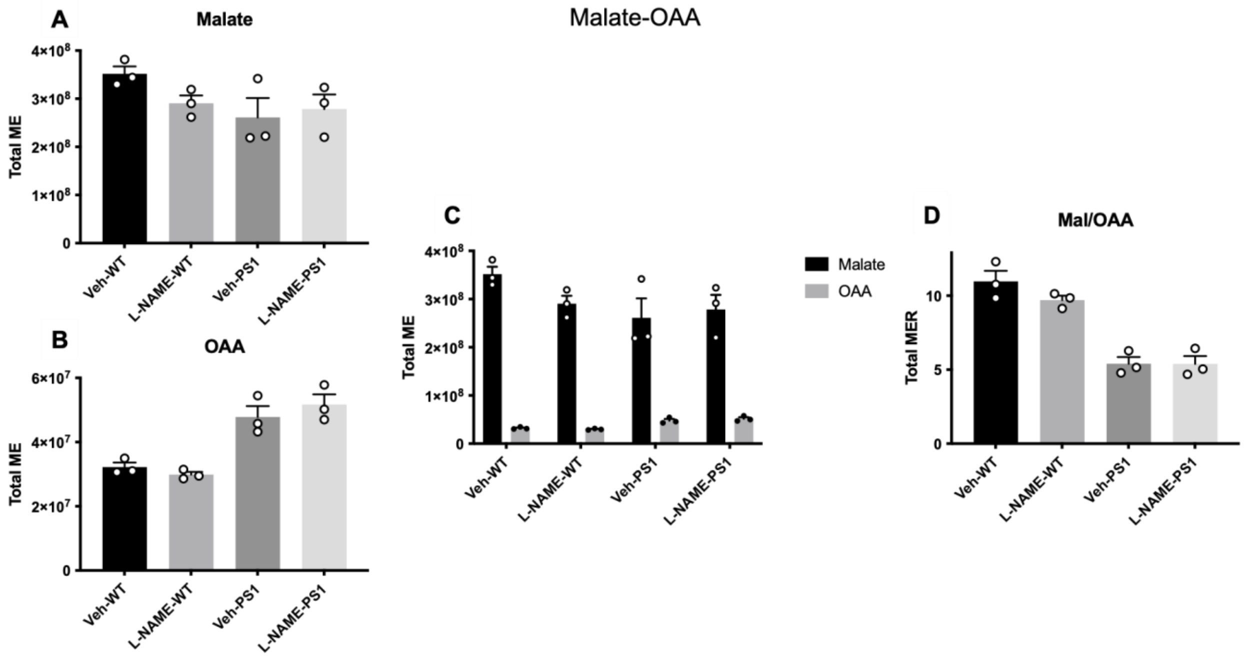

Figure S5. Total ME and MER data for the pair of metabolites malate and oxaloacetate (OAA), representing flux through malate dehydrogenase

Values are mean + SEM (n = 3).

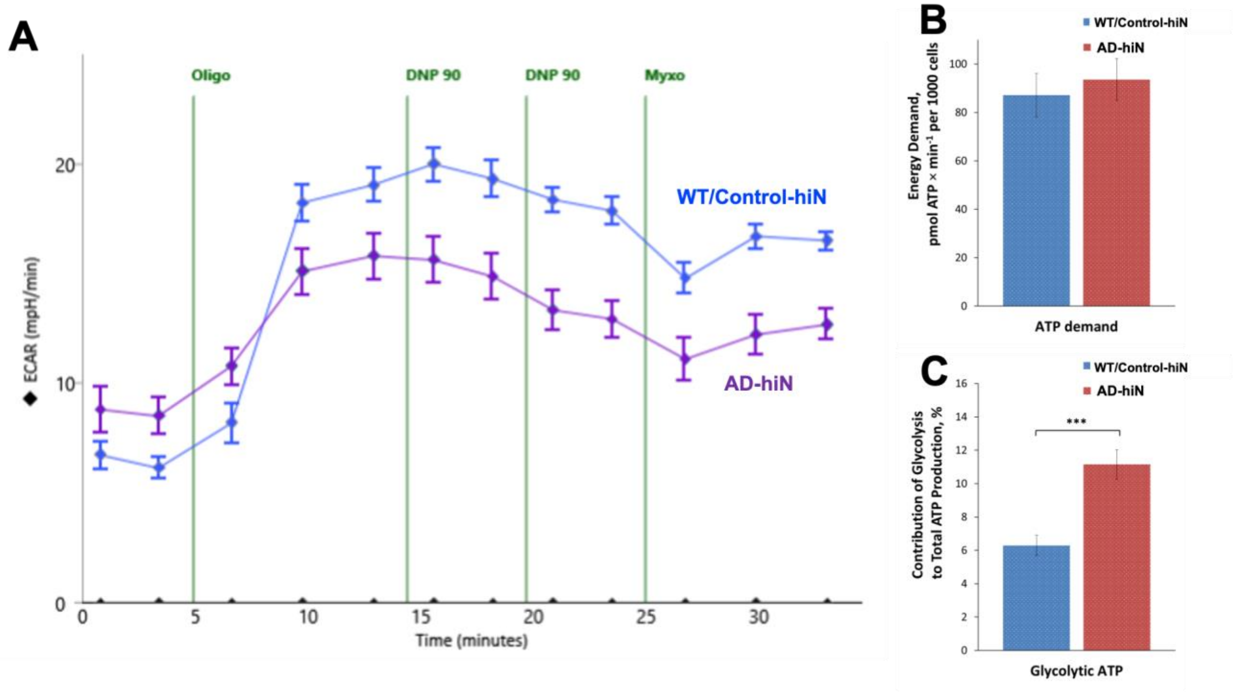

Figure S6. Compensatory activation of aerobic glycolysis in respiratory-deficient AD-hiN (Warburg-like effect)

(**A**) Representative experiment in Seahorse Flux Analyzer showing extracellular acidification rate (ECAR) for PS1 AD-hiN vs. WT/Control hiN. Injections are shown by vertical lines: Oligo, 2 μg/ml oligomycin; two injections of DNP, 90 μM; Myxo, 2 μM myxothiazol.

(**B, C**) Increased contribution of glycolysis to total ATP production in PS1 AD-hiN vs. WT/Control-hiN. Total ATP production in the basal state of hiN, reflecting cellular energy demand, is unaffected by genotype (**B**). However, contribution of glycolysis to total ATP production increases about 2-fold in AD-hiN compared to WT/Control hiN (**C**), thus compensating in part for the decrement in ATP observed during oxidative phosphorylation (OXPHOS) in AD-hiN (see **Figure S7**). Data are mean± SEM; ***p < 0.001, by Student's t-test (n = 22 plates analyzed, each in separate experiments for panel B; n = 43 plates for panel C).

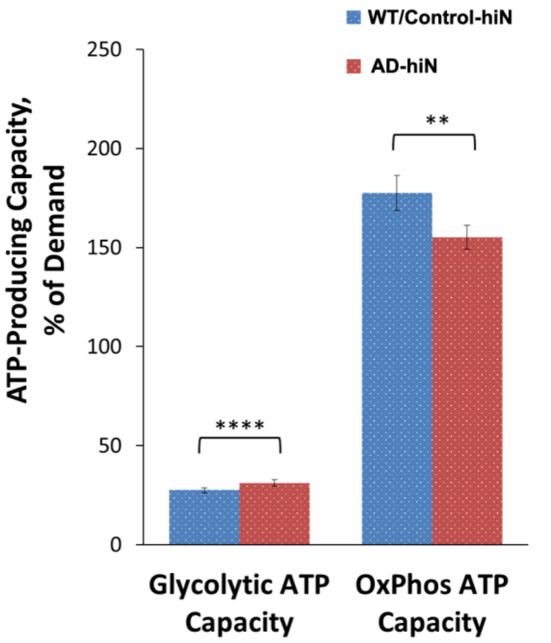

Figure S7. Deficiency in spare energy-transducing capacity in PS1 AD-hiN neurons compared to WT/Control-hiN

Calculated ATP-producing capacity (see METHOD DETAILS) for glycolysis (bars on *left*) and oxidative phosphorylation (OXPHOS, TCA cycle plus ETC) (bars on *right*) relative to total ATP demand (as shown in **Figure S6B**). Maximally-stimulated glycolysis by itself is incapable of meeting energy demands of hiN reaching only about 30-40% of required ATP production. OXPHOS possesses spare respiratory capacity in WT/Control-hiN which, however, is suppressed in AD-hiN (reaching ~170% in WT/Control-hiN but only ~150% in AD-hiN). Data are mean ± SEM; $^{**}p < 0.01$, $^{****}p < 0.0001$ by Student's t-test (n = 43 plates tested, each in a separate experiment).

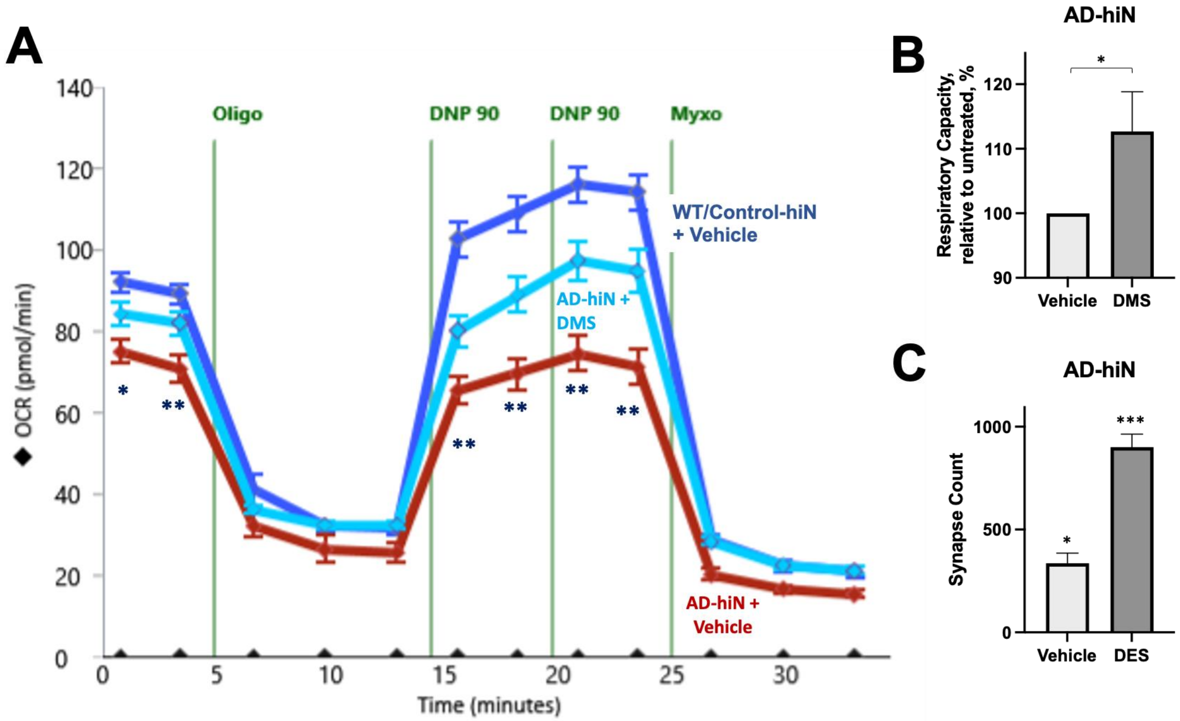

Figure S8. Respiratory defect in APP^{Swe} AD-hiN compared to WT/Control-hiN with partial rescue with cell-permeable succinate derivatives of OCR and synapse number

(**A**) Representative experimental run in Seahorse Flux Analyzer with 8-week terminally differentiated APP^{Swe} AD-hiN and WT/Control-hiN (10 wells per experimental group). OCR, oxygen consumption rate. Injections shown by vertical lines: Oligo, 2 µg/ml oligomycin; DNP, 120 µM 2,4-dinitrophenol; Myxo, 2 µM myxothiazol; DMS, 5 mM dimethyl succinate added 20 min prior to the run.

(**B**) Respiratory capacity representing maximal uncoupler-induced OCR per 1,000 cells attained after 4 sequential additions of the uncoupler DNP. Data are mean ± SEM determined in replicate cultures of pure neurons (n = 10 wells per group in a single plate of hiN per experiment, with data from 17 separate experiments obtained in separate hiPSC differentiations. *p < 0.05 by Student's t-test. For these experiments, DMS = 0.5 to 5 mM administered for 20 min to 4 hr.

(**C**) Treatment with diethyl succinate (DES, 5 mM, an even more cell-permeant analogue of succinate than DMS) for 48 hr led to partial recovery of synaptic number relative to untreated

APP^{Swe} AD-hiN. Data are mean ± SEM of total synapses imaged by concordance of pre- and postsynaptic markers by confocal microscopy (n = 6 wells of a 96-well plate per group monitored at 5 sites per well); *$p < 0.05$ compared to WT; ***$p < 0.001$ for DES compared to vehicle by Student's t-test. Note for APP^{Swe} AD-hiN, the WT/Control is the same isogenic line used for the PS1 AD-hiN, and these data are shown in Figure 4.

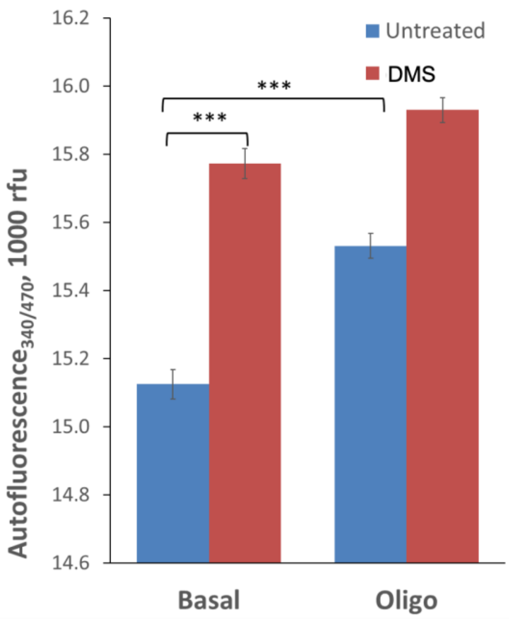

Figure S9. Autofluorescence assay of relative mitochondrial NADH levels in hiN

Effect of dimethyl succinate (DMS, 5mM) on reductive status of mitochondrial pyridine nucleotides in PS1 AD-hiN. Autofluorescence of pyridine nucleotides (yielding relative NADH levels) was measured as described in METHOD DETAILS. Data are mean and SEM of intensity of individual mitochondrial puncta (n = 200 per field), shown as relative fluorescence units (rfu at 340/470 nm excitation/emission); ***$p < 0.001$ by Student's t-test. Additions: Untreated for respiratory basal state; Oligo, 2 µg/ml oligomycin for resting state (State 4).

Table S1. Data from the organomercury-chemoselective enrichment/MS platform

(Tab 1) EXCEL spreadsheet labeled "Human Brains Information" has Demographics of human AD and control brains analyzed.

(Tabs 2 and 3) EXCEL spreadsheets labeled "Control" and "AD" contain SNO-sites found on proteins by MS in Control and AD brains, respectively.

(Tabs 4 and 5) EXCEL spreadsheets, labeled "Unique to Control" and Unique to AD" show SNO-sites on proteins found only in Control of AD brains, respectively.

(Tabs 6-8) EXCEL spreadsheets labeled "Shared Proteins and Sites," "Shared Proteins New Sites Control," and "Shared Proteins New Sites AD" contain lists of proteins with identical S-nitrosylated proteins and SNO-sites, shared S-nitrosylated proteins but with additional SNO-sites found only in Control brains, and shared S-nitrosylated proteins but with additional SNO-sites only in AD brains, respectively.

(Tab 9) EXCEL spreadsheet labeled "Venn Data Summary" illlustrates numbers of S-nitrosylated proteins and peptides found in Control human brains, AD human brains, and shared between them.

(Tab 10) EXCEL spreadsheet labeled "Shared Sites $p < 0.05$" shows S-nitrosylated proteins and their SNO-sites that are significantly ($p < 0.05$) up or downregulated in Control and AD human brains.

(Tab 11) EXCEL spreadsheet labeled "TCA Enzymes" contains a list of S-nitrosylated enzymes related to the TCA cycle and their SNO-sites and whether they are differentially S-nitrosylated in AD compared to Control human brains.

(Tab 12) EXCEL spreadsheet labeled "GO_BP_CONTROL" shows a list of GO biological process terms (with fold enrichment and false discovery rate [FDR]) that manifest S-nitrosylated proteins from Control human brains.

(Tab 13) EXCEL spreadsheet labeled "GO_BP_CONTROL" shows a list of GO biological process terms (with fold enrichment and false discovery rate [FDR]) that manifest S-nitrosylated proteins from AD human brains.
